# 1,3,2,5-Diazadiborinine featuring nucleophilic and electrophilic boron centres

**DOI:** 10.1038/ncomms8340

**Published:** 2015-06-15

**Authors:** Di Wu, Lingbing Kong, Yongxin Li, Rakesh Ganguly, Rei Kinjo

**Affiliations:** 1 Division of Chemistry and Biological Chemistry, Nanyang Technological University, 21 Nanyang Link, 637371 Singapore, Singapore; 2 NTU-CBC Crystallography Facility, Nanyang Technological University, 21 Nanyang Link, 637371 Singapore, Singapore

## Abstract

The seminal discovery in 1865 by Kekulé that benzene nucleus exists with cyclic skeleton is considered to be the beginning of aromatic chemistry. Since then, a myriad of cyclic molecules displaying aromatic property have been synthesized. Meanwhile, borazine (B_3_N_3_H_6_), despite the isostructural and isoelectronic relationships with benzene, exhibits little aromaticity. Herein, we report the synthesis of a 1,3,2,5-diazadiborinine (B_2_C_2_N_2_R_6_) derivative, a hybrid inorganic/organic benzene, and we present experimental and computational evidence for its aromaticity. In marked contrast to the reactivity of benzene, borazine, and even azaborinines previously reported, 1,3,2,5-diazadiborinine readily forms the adducts with methyl trifluoromethanesulfonate and phenylacetylene without any catalysts. Moreover, 1,3,2,5-diazadiborine activates carbon dioxide giving rise to a bicycle[2,2,2] product, and the binding process was found to be reversible. These results, thus, demonstrate that 1,3,2,5-diazadiborinine features both nucleophilic and electrophilic boron centres, with a formal B(+I)/B(+III) mixed valence system, in the aromatic six-membered B_2_C_2_N_2_ ring.

## Abstract (web summary)

Boron atoms in aromatic heterocycles typically behave simply as electron pair acceptors. Here, the authors report an aromatic system containing both electrophilic and nucleophilic boron atoms, and demonstrate how this imparts frustrated Lewis pair type reactivity, including binding of carbon dioxide.

## Graphical abstract

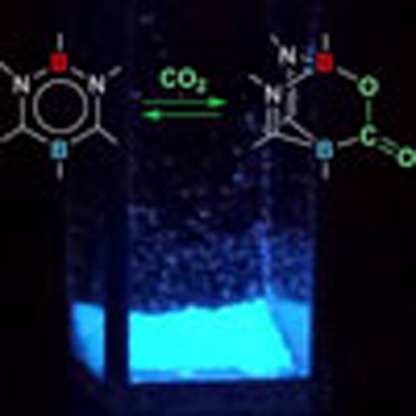

The concept of aromaticity has been of paramount importance in myriad fields of chemistry since the discovery in 1865 that benzene nucleus is cyclic^1^. Almost a century after the first identification of benzene by Faraday^2^, borazine (B_3_N_3_H_6_), also referred as inorganic benzene, was prepared^3^. Despite the isoelectronic and isosterism relationships between the C=C and B–N units, however, B_3_N_3_H_6_ displays a different electronic property from that of benzene^4^,^5^, which is due to polarization of the B–N units arising from the variation of electronegativity between boron and nitrogen atoms. Thus, introduction of B–N units into the aromatic skeleton of isoelectronic organic counterparts leads to unique electronic structures, which indicate the potential to expand the diversity of aromatic molecules^6^,^7^,^8^.

In 1958, Dewar and co-workers first reported the preparation of a polycyclic azaborinine^9^. Since then, the synthesis and full characterization of various mono-cyclic and ring-fused polycyclic azaborinine (BNC_4_) derivatives, involving 1,2-azborinines, 1,3-azaborinines and 1,4-azaborinines, have been achieved^10^. Meanwhile, only a few diazadiborinine (B_2_N_2_C_2_) derivatives have been structurally characterized so far^11^,^12^. Thermal stability as well as aromatic nature of both azaborinine and diazadiborinine more closely resembles those of benzene than B_3_N_3_H_6_. Nevertheless, except for *η*^6^-complexation with a chromium and ruthenium metals, the reactivities of the boron centre in these compounds are mainly associated with nucleophilic substitutions, which is in contrast to the reactivity of benzene where electrophilic substitution is archetypal. Thus, the boron in these compounds only acts as a classical electron pair acceptor. Recently, Bertrand and our groups^13^,^14^,^15^,^16^ independently developed neutral tricoordinate organoboron species possessing a nucleophilic boron centre, which is formally in the +I oxidation state. We were interested in incorporating a nucleophilic boron centre into diazadiborinine skeleton because the resulting B_2_N_2_C_2_ ring would involve both nucleophilic and electrophilic boron centres, which can be formally considered as a B(+I)/B(+III) mixed valence system^17^,^18^,^19^,^20^. Among extant non-metal-based mixed valence compounds^21^,^22^, it has been reported that charge-neutral mixed valence system especially with closed-shell form, namely donor–acceptor system, exhibit high stability^23^. We reasoned that incorporation of the mixed valence system into aromatic ring would effectively lead to charge delocalization as found in mixed valence bimetallic compounds categorized into class II and III^24^,^25^. Meanwhile, preparation of such molecules with p-block heteroatoms is highly challenging owing to the limitation of synthetic approach for their low oxidation state. Indeed, for heterobenzene featuring mixed valence systems of the p-block inorganic elements, only the valence isomers of hexasilabenzene^26^ and tetraphosphabenzene^27^ are known. No relevant species involving mixed valence system of boron atoms have been described thus far.

Herein, we report the synthesis, single-crystal X-ray diffraction analysis and computational studies of 1,3,2,5-diazadiborinine **4**. We show that this compound possesses both nucleophilic and electrophilic boron centres with a formal B(+I)/B(+III) mixed valence system in the aromatic six-membered ring.

## Results

### Synthesis and characterization of 4

**Figure 1:**
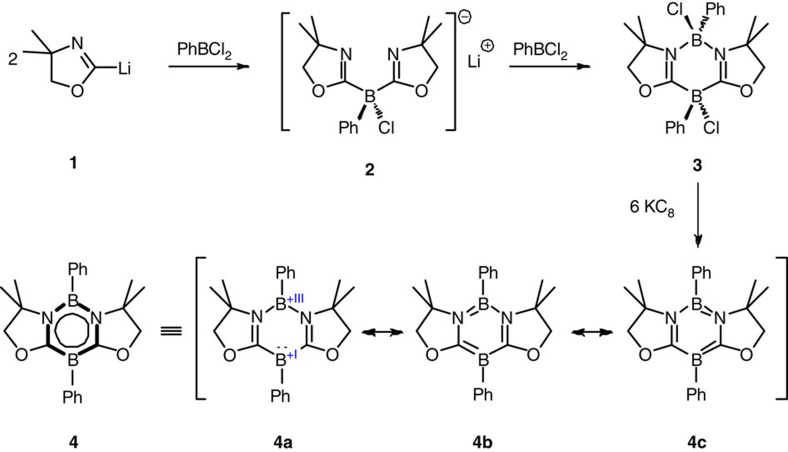
Preparation of 1,3,2,4-diazadiborinine **4**. 4a–c present the resonance forms.

Oxazolinyl groups were introduced into a boron atom by treatment of two equivalents of 2-lithio-4,4'-dimethyl-2-oxazolide **1** with dichlorophenylborane (Fig. 1). Without further purification of the crude product **2**, a subsequent reaction with one equivalent of dichlorophenylborane in toluene afforded a 2,5-dichloro-1,3,2,5-diazadiborinine derivative **3** (29% yield), which was fully characterized by standard spectroscopic methods, including a single-crystal X-ray diffraction study. Treatment of **3** with excess amounts of potassium graphite (KC_8_) in toluene cleanly proceeded, and after workup 1,3,2,5-diazadiborinine derivative **4** was isolated as a white powder in 32% yield. In the ^11^B nuclear magnetic resonance (NMR) spectrum of **4**, a sharp signal for the boron atom between two carbon atoms appeared at *δ*=7.3 parts per million (p.p.m.) and a broad peak for the boron atom between two nitrogen atoms was observed at *δ*=24.9 p.p.m. Both signals shifted downfield compared with those of the precursor **3** (*δ*=−11.4 and 3.5 p.p.m.). Compound **4** is thermally stable both in the solid state and in solutions, and it melts at 133 °C without decomposition.

**Figure 2:**
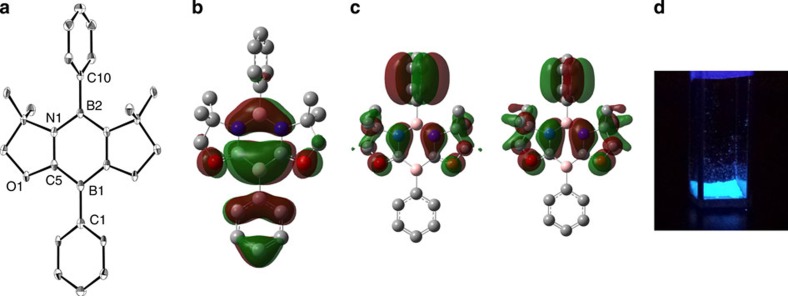
Structural characterization and fluorescence property. (**a**) Solid state structure of **4**. Thermal ellipsoids are set at the 30% probability level. Hydrogen atoms are omitted for clarity. (**b**) Plot of the HOMO of **4**. (**c**) Plots of the HOMO-4 (left) and HOMO-5 (right) of **4**. Calculated at the B3LYP/6-311+G(d,p) level of theory. Hydrogen atoms are omitted for clarity. (**d**) Photographic image of fluorescence emission in the solid state of **4** under irradiation of a ultraviolet lamp.

Single crystals of **4** suitable for X-ray diffractometry were obtained by recrystallization from a benzene solution at room temperature, and crystallographic analysis revealed that the six-membered B_2_C_2_N_2_ ring of **4** is nearly planar (Fig. 2a). Two boron atoms display trigonal-planar geometry (the sum of bond angles: B1=359.96° and B2=359.93°), which are characteristic for *sp*^2^ hybridization. Phenyl ring at B2 and the B_2_C_2_N_2_ six-membered ring are nearly perpendicular to each other with the twist angle of 89.1°, whereas phenyl group at B1 and the B_2_C_2_N_2_ skeleton are slightly twisted by 11.9°. The B1–C5 (1.483(3) Å) and B2–N1 (1.443(3) Å) distances are significantly shorter than those (1.575(5)–1.589(5) Å and 1.573(4)–1.563(4) Å) in **3**, and lie between typical single and double-bond distances of boron–carbon and boron–nitrogen bonds, respectively. In contrast, the N1–C5 distance of 1.374(3) Å is longer than that (1.287(4)–1.292(4) Å) in **3**. These structural features suggest the delocalization of 6π-electrons over the six-membered B_2_C_2_N_2_ ring in **4**, which can be represented by the average of the several canonical forms including **4a**–**c**.

### Computational studies

To gain further insight into the electronic features of **4**, quantum chemical density functional theory calculation involving geometry optimization, natural bond orbital analysis and natural population analysis were performed at the B3LYP/6-311G+(d,p) level of theory. The optimized geometry of **4** was in good agreement with the structural parameters determined experimentally. Natural bond orbital analysis gave Wiberg bond index value for the boron–nitrogen bonds (0.96 for B2–N1). Meanwhile, Wiberg bond index values lager than 1 for the B1–C5 bonds (1.21) and the C5–N1 bonds (1.14) were obtained, thus suggesting the partial double-bond character of these bonds. Indeed, the HOMO of **4** displays a π-system over the six-membered B_2_C_2_N_2_ ring featuring a node between the NBN and CBC π-unites, which exhibits anti-bonding conjugation with the π-orbital in the phenyl ring bounded to the B1 atom (Fig. 2b). π-Bonding interactions between the C5 atom and the N1 atom were confirmed in HOMO-4 and HOMO-5 (Fig. 2c). In the ultraviolet–visible absorption spectrum of **4** in a tetrahydrofuran (THF) solution, an absorption band was observed at wavelength (*λ*) of 275 nm, which is comparable to that (*λ*=277 nm) of *para*-terphenyl (PhC_6_H_4_Ph) bearing all-carbon aromatic skeleton isoelectronic with **4** (ref. 28). Interestingly, we observed that **4** has little fluorescence in any solution, whereas it displays distinct light blue-fluorescence emission in the solid state under a ultraviolet lamp (Fig. 2d; refs 29, 30). The broad emission peak of **4** at (*λ*=423 nm) was bathochromically shifted by 80 nm in comparison with *para*-terphenyl (*λ*=343 nm).

**Figure 3:**
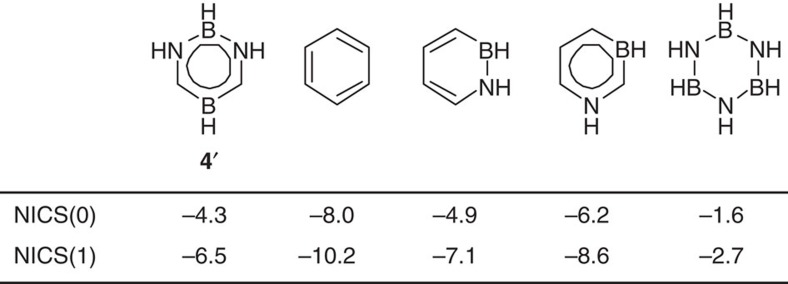
Results of density functional theory calculations. Calculated NICS(0) and NICS(1) values for **4**′, benzene, 1,2-azaborine, 1,3-azaborine and B_3_N_3_H_6_. Calculated at the B3LYP/6-311+G(d,p) level of theory.

To evaluate the aromatic property of **4**, the nucleus-independent chemical-shift values NICS(0) and NICS(1) were calculated for parent 1,3,2,5-diazadiborinine **4**′, benzene (C_6_H_6_), 1,2-azaborine, 1,3-azaborine and B_3_N_3_H_6_ (Fig. 3). The NICS values for **4**′ are less negative than those of benzene and 1,3-azaborine, but comparable to those of 1,2-azaborine, and more negative with respect to those of B_3_N_3_H_6_. Thus, it is predicted that 1,3,2,5-diazadiborinine features aromaticity, which seems smaller than those of benzene and 1,3-azaborine, but greater than that of B_3_N_3_H_6_. To estimate the resonance stabilization energy (RSE) of the parent 1,3,2,5-diazadiborinine **4**′, we performed further computational analysis. The RSE value of **4**′ is ∼22.4 kcal mol^−1^ less than that of benzene (34.1 kcal mol^−1^), which is smaller than those of 1,3-azaborinine (RSE=29 kcal mol^−1^) (ref. 31) and 1,2-azaborinine (RSE=21 kcal mol^−1^) (ref. 32).

### Reactivity

**Figure 4:**
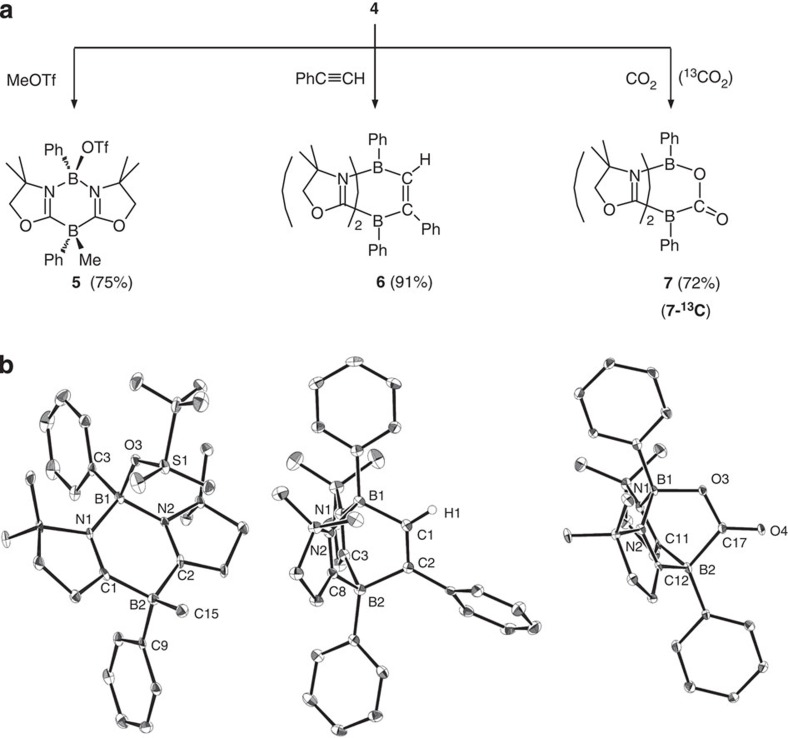
Reactivity of 4. (**a**) Reactions of **4** with MeOTf, PhC≡CH and CO_2_ (^13^CO_2_). (**b**) Solid state structures of **5** (left), **6** (middle) and **7** (right). Thermal ellipsoids are set at the 30% probability level. Hydrogen atoms except for H1 in **6** and solvent molecules are omitted for clarity.

To investigate the reactivity of **4**, we performed its reaction with methyl trifluoromethanesulfonate (MeOTf). A stoichiometric amount of MeOTf was added to an acetonitrile solution of **4** at ambient temperature. After removing the solvent under vacuum, **5** was obtained in 75% yield (Fig. 4a). An X-ray diffraction study confirmed that methyl group is attached to the boron atom between two carbons in the B_2_C_2_N_2_ ring, whereas an oxygen atom of the triflate is bounded to the boron atom between two nitrogen atoms (Fig. 4b, left). This result, thus, demonstrates that **4** features both nucleophilic and electrophilic boron centres, thereby supporting the electronic property of the resonance structure **4a** (Fig. 1). The formal oxidation states of the B1 and the B2 atoms in **4a** are +I and +III, respectively. Thus, **4** presents a donor–acceptor mixed valence system.

Reactivity of neutral boron nucleophiles has seldom been explored thus far^13^,^14^,^15^,^16^. Based on the behaviours of **4** with MeOTf, we postulated that **4** would act as a frustrated Lewis pair, since it possesses both Lewis basic and acidic boron centres^33^,^34^,^35^,^36^,^37^. To bear out the hypothesis, we next investigated the reactivity of **4** towards non-activated alkynes. In a J-Young NMR tube, a stoichiometric amount of phenylacetylene was added to a C_6_D_6_ solution of **4**, and reaction was monitored by NMR spectroscopy. After 2 h at 70 °C, two new signals were detected at −13.6 and 0.1 p.p.m. in the ^11^B NMR spectrum. After workup, compound **6** was isolated as a white powder in 91% yield. The crystallographic study revealed the diazadiborabicycle[2.2.2] structure involving the C=N double bonds (N1–C3: 1.299(4) Å and N2–C8: 1.299(3) Å) (Fig. 4b, middle). Thus, **6** is a formal Diels–Alder product via a [4+2] cyclo-addition between **4** and the carbon–carbon triple bond of phenylacetylene. It is noteworthy to mention that Diels–Alder addition of alkynes to aromatic hydrocarbons normally is restricted to highly reactive aromatic compounds and activated alkynes under harsh conditions^38^,^39^. Moreover, **6** can be viewed as an analogue of bicycle[2.2.2]octatriene, also termed barrelene, which is inferred to be one of the Möbius aromatic compounds^39^.

Next, we examined the carbon dioxide (CO_2_) activation with **4** (ref. 40). CO_2_ gas was introduced into a benzene solution of **4** at 1 bar, and the solution was heated at 70 °C. After 2 h, a white precipitate was filtered and dried under vacuum to afford compound **7** in 72% yield. We also carried out a ^13^C-labelling study using ^13^CO_2_, which produced **7-**^**13**^**C** (80% yield). The ^13^C NMR spectrum of **7-**^**13**^**C** displayed a broad resonance at 191.1 p.p.m. In the ^11^B NMR spectrum, a set of new peaks was observed at 3.1 p.p.m. as a broad singlet and −16.7 p.p.m. as a broad doublet (^1^*J*_B-C_=42.4 Hz), which is owing to the coupling with a ^13^C carbon atom. These results indicate the presence of a bond between a boron and the carbon from CO_2_ in **7**, which was decisively confirmed by X-ray diffractometry (Fig. 4b, right). One of the C=O double bonds of CO_2_ was cleaved and new B–C and B–O bonds are formed through 1,4 addition, which is contrast to the behaviour of benzene that reacts with CO_2_ to form benzoic acid only in the presence of Lewis acid catalysts^41^. We also found that the CO_2_ activation process by **4** was reversible. Thus, treatment of **7** at 90 °C for 50 min reproduced **4**, quantitatively.

## Discussion

One-hundred fifty years after the discovery of the cyclic structure of benzene, 1,3,2,5-diazadiborinine derivative **4** featuring a formal B(+I)/B(+III) mixed valence system has joined as a heteroanalogue into a class of 6π-Hückel aromatic molecules. In marked contrast to the chemical behaviour of benzene, B_3_N_3_H_6_ or even other azaborinines, **4** exhibits unique optical property and reactivity like a boron–boron frustrated Lewis pair. Because the electronic property can be substantially modulated by varying the substituents on each boron atom, the isolation of this molecule paves the way for the discovery of new materials with useful photochemical properties. Moreover, the cooperative reactivity of the nucleophilic and electrophilic boron centres, and the reversible nature for activation of small molecules will be applicable to catalytic chemistry.

## Methods

### Materials

For details of spectroscopic analyses of compounds in this manuscript, see Supplementary Figs 1–25. For details of density functional theory calculations, see Supplementary Fig. 26, Supplementary Tables 3–5 and Supplementary Methods. For details of the synthetic procedures, see Supplementary Methods. For details of X-ray analysis, see Supplementary Data 1–5, Supplementary Tables 1 and 2, and Supplementary Methods.

### General synthetic procedures

All reactions were performed under an atmosphere of dry argon using standard Schlenk or dry box techniques; solvents were dried over Na metal, K metal or CaH_2_, and were distilled under nitrogen. Reagents were of analytical grade, obtained from commercial suppliers and were used without further purification. ^1^H, ^13^C, ^11^B and ^19^F NMR spectra were recorded on a Bruker AVIII 400 MHz or Bruker Avance 500 MHz AV500 spectrometers at 298 K. Chemical shifts (*δ*) are given in p.p.m. Coupling constants *J* are given in Hz. In the ^13^C NMR spectra of compounds **3**–**7**, presumable owing to the coupling with boron atoms, signals for the carbon atoms directly bonding to boron atoms could not be observed. Electrospray ionization (ESI) mass spectra were obtained at the Mass Spectrometry Laboratory at the Division of Chemistry and Biological Chemistry, Nanyang Technological University. Melting points were measured with OptiMelt (Stanford Research System). Fourier-transform infrared (FT-IR) spectra were recorded on a SHIMADZU IRPrestige-21 spectrometer using solid compound. Ultraviolet and fluorescence spectra were recorded on Cary 100 UV-Vis and SHIMADZU RF-5301PC spectrofluorophotometer, respectively.

### Synthesis of 3

A hexane solution (1.6 M) of *n*-BuLi (6.25 ml, 10.00 mmol) was added dropwise into a THF solution (50 ml) of 4,4-dimethyl-2-oxazoline (1.00 ml, 9.48 mmol) at –78 °C. After stirring for 1 h at –78 °C, dichlorophenylborane (0.62 ml, 4.72 mmol) was added into the solution. The reaction mixture was warmed to room temperature and stirred overnight. After the solvent was removed under vacuum, toluene (60 ml) was added and salts were filtered off, which was used directly for next step without further purification. Dichlorophenylborane (0.62 ml, 4.72 mmol) was added dropwise into the solution at –78 °C. The reaction mixture was left to stir for 1 h at –78 °C, and slowly warmed to room temperature and stirred overnight. The solvent was removed under vacuum, and the solid residue was recrystallized from benzene to afford colourless crystals of **3** (0.61 g, 29%). Melting point (Mp): 207 °C. ^1^H NMR (400 MHz, C_6_D_6_): *δ*=0.52 (s, 6 H), 1.21 (s, 6 H), 3.06 (d, 2H, *J*=9.2 Hz), 3.13 (d, 2 H, *J*=8.8 Hz), 7.21–8.29 (m, 10 H); ^13^C NMR (100 MHz, C_6_D_6_):*δ*=24.2, 27.5, 68.3, 81.8, 127.6, 132.4, 133.1, 136.0; DEPT—135 NMR (100 MHz, C_6_D_6_): *δ*=24.2, 27.5, 127.6, 128.0, 128.3. 132.4, 133.1, 136.0. ^11^B NMR (76.8 MHz, C_6_D_6_): *δ*=–11.4 (s), 3.5 (s); HRMS (ESI): *m/z* calculated for C_22_H_26_B_2_N_2_O_2_Cl: 407.1869 [(*M*)]^+^; found: 407.1866.

### Synthesis of 4

Potassium graphite (1.49 g, 11.04 mmol) was slowly added to a solution of **3** (0.82 g, 1.84 mmol) in toluene (50 ml) and stirred overnight at room temperature. After filtration, the solvent was concentrated to 5 ml under vacuum. The solid residue was filtered off and washed with hexane (15 ml), and then dried under vacuum to afford **4** as a white powder (0.22 g, 32%). Mp: 133 °C. ^1^H NMR (400 MHz, C_6_D_6_): *δ*=0.79 (s, 12 H), 3.63 (s, 4 H), 7.09–8.84 (m, 10 H); ^13^C NMR (100 MHz, C_6_D_6_): *δ*=27.5, 62.8, 80.9, 125.4, 127.1, 127.7, 134.7, 135.1; DEPT—135 NMR (100 MHz, C_6_D_6_): *δ*=27.5, 125.4, 127.1, 127.7, 128.4, 134.7, 135.1; ^11^B NMR (76.8 MHz, C_6_D_6_): *δ=*7.3 (s), 24.9 (s); Ultraviolet–visible (THF): *λ*=275 nm (*ɛ*: 5,740); HRMS (ESI): *m/z* calculated for C_22_H_27_B_2_N_2_O_2_: 373.2259 [(*M+H*)]^+^; found: 373.2271; IR (KBr, cm^−1^): 1591.3, 1475.5, 1444.7, 1421.5, 1392.6, 1375.3, 1303.9, 1253.7, 1205.5, 1103.3, 1074.4, 1033.9, 1016.5, 954.8, 939.3.

### Reaction of 4 with MeOTf

MeOTf (7.42 μl, 0.068 mmol) was added into an acetonitrile (6 ml) solution of **4** (0.021 g, 0.056 mmol) and stirred for 1 h at room temperature. After the solvent was removed under vacuum, the solid residue was recrystallized from a 2:1 mixture of fluorobenzene/hexane to afford colourless crystals of **5** (0.022 g, 75%). Mp: 151 °C (dec). ^1^H NMR (400 MHz, THF-d_8_): *δ*=0.34 (s, 3 H), 0.87 (s, 6 H), 1.38 (s, 6 H), 4.14 (d, 2 H, *J*=8.8 Hz), 4.26 (d, 2 H, *J*=8.4 Hz), 7.06–7.66 (m, 10 H); ^13^C NMR (100 MHz, THF-d_8_): *δ*=22.4, 29.0, 67.3, 83.2, 126.1, 127.7, 128.1, 129.6, 130.8, 132.4; ^11^B NMR (76.8 MHz, THF-d_8_): *δ*=–19.7 (s), 4.2(s); ^19^F NMR (225.6 MHz, THF-d_8_): *δ*=–81.2; HRMS (ESI): *m/z* calculated for C_24_H_30_B_2_N_2_O_5_SF_3_: 537.2014 [(*M+H*)]^+^; found: 537.2006.

### Reaction of 4 with phenylacetylene

Phenylacetylene (9.2 μl, 0.087 mmol) was added into a C_6_D_6_ (0.5 ml) solution of **4** (0.031 g, 0.083 mmol) and heated at 70 °C. Reaction was monitored by NMR spectroscopy. After 2 h, the solvent was removed under vacuum, and the solid residue was recrystallized from THF/hexane to afford colourless crystals of **6** (0.036 g, 91%). Mp: 120 °C. ^1^H NMR (400 MHz, C_6_D_6_): *δ*=0.52 (s, 6 H), 0.87 (s, 6 H), 3.44 (d, 2 H, *J*=8.4 Hz), 3.51 (d, 2 H, *J*=8.4 Hz), 7.01–8.04 (m, 16 H); ^13^C NMR (125 MHz, CDCl_3_): *δ*=25.0, 27.4, 65.9, 84.9, 124.7, 125.3, 126.7, 127.2, 127.6, 127.9, 128.5, 132.3, 135.2; ^11^B NMR (76.8 MHz, C_6_D_6_): *δ*=–13.6 (s), 0.1 (s); HRMS (ESI): *m/z* calculated for C_30_H_33_B_2_N_2_O_2_: 475.2728 [(*M+H*)]^+^; found: 475.2733.

### Reaction of 4 with CO_2_

A benzene (15 ml) solution of compound **4** (0.061 g, 0.040 mmol) was degassed using a freeze-pump-thaw method, and then CO_2_ (1 bar) was introduced into the schlenk tube at room temperature. After 2 h at 70 °C, a white precipitate was collected by filtration and dried under vacuum to afford **7** (0.049 g, 72%). Mp: 158 °C (dec). ^1^H NMR (400 MHz, CD_2_Cl_2_): *δ*=0.90 (s, 6 H), 1.50 (s, 6 H), 4.36 (d, 2 H, *J*=9.2 Hz), 4.39 (d, 2 H, *J*=9.2 Hz), 7.22–7.95 (m, 10 H); ^13^C NMR (100 MHz, CD_2_Cl_2_): *δ*=25.9, 27.7, 66.5, 86.4, 126.9, 127.5, 129.1, 134.97, 134.99, 135.1; ^11^B NMR (76.8 MHz, CD_2_Cl_2_): *δ*=–16.7 (s), 3.1 (s); HRMS (ESI): *m/z* calculated for C_23_H_27_B_2_N_2_O_4_: 417.2157 [(*M+H*)]^+^; found: 417.2161.

### Reaction of 4 with ^13^CO_2_

Compound **4** (0.015 g, 0.040 mmol) was added to a sealable J-Young NMR tube and was dissolved in C_6_D_6_ (0.35 ml). The sample was degassed using a freeze-pump-thaw method. Labile ^13^CO_2_ was condensed into the NMR tube at 77 K, followed by warming to room temperature, and reacted for 2 h at 70 °C. A white precipitate was collected by filtration and dried under vacuum to afford **7-**^**13**^**C** (0.013 g, 80% yield). ^13^C NMR (100 MHz, CD_2_Cl_2_): *δ*=25.9, 27.7, 66.5, 86.4, 126.9, 127.5, 129.1, 134.97, 134.99, 135.1, 191.1 (br d, *J*_CB_=42.4 Hz); ^11^B NMR (76.8 MHz, CD_2_Cl_2_): *δ*=–16.7 (br d, *J*_BC_=42.4 Hz), 3.1 (s).

### Regeneration of 4 from 7

Compound **7** was dissolved in a 1:1 mixture of CD_3_CN/THF-d_8,_ and heated at 90 °C. Reaction was monitored by ^1^H and ^11^B NMR spectroscopy and after 50 min, a quantitative regeneration of **4** was confirmed.

## Additional information

**Accession codes:** The X-ray crystallographic coordinates for structures reported in this article have been deposited at the Cambridge Crystallographic Data Centre (CCDC), under deposition numbers CCDC 1049275–1049279. These data can be obtained free of charge from The Cambridge Crystallographic Data Centre via www.ccdc.cam.ac.uk/data_request/cif.

**How to cite this article**: Wu, D. *et al.* 1,3,2,5-Diazadiborinine featuring nucleophilic and electrophilic boron centres. *Nat. Commun.* 6:7340 doi: 10.1038/ncomms8340 (2015).

## Supplementary Material

Supplementary Information Supplementary Figures 1-26, Supplementary Tables 1-5, Supplementary Methods and Supplementary References

Supplementary Data 1 Crystallographic information for compound 3

Supplementary Data 2 Crystallographic information for compound 4

Supplementary Data 3 Crystallographic information for compound 5

Supplementary Data 4 Crystallographic information for compound 6

Supplementary Data 5 Crystallographic information for compound 7
