# Supplementary material for: 1,3,2,5-Diazadiborinine featuring nucleophilic and electrophilic boron centres
Source: Nat Commun. 2015 Jun 15;6:7340. doi: 10.1038/ncomms8340 (PMC4490389; doi:10.1038/ncomms8340)
Supplement: Supplementary Information — Supplementary Figures 1-26, Supplementary Tables 1-5, Supplementary Methods and Supplementary References [file ncomms8340-s1.pdf]

Supplementary Figures

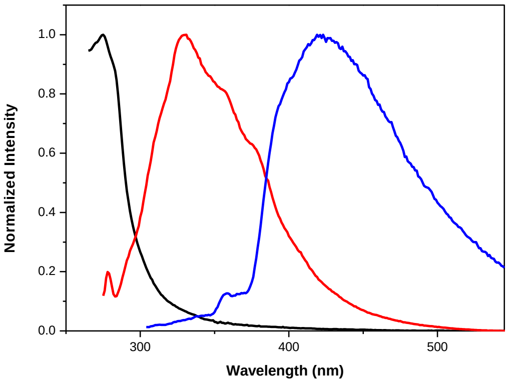

Supplementary Figure 1 | The *normalized* absorption in THF (black line) and fluorescence spectra in THF (red line) and in the solid-state (blue line) of **4**.

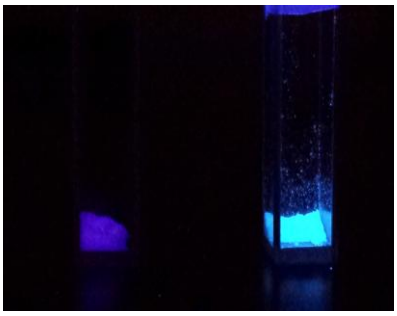

Supplementary Figure 2 | The image of **4** (right) and *para*-terphenyl (PhC_6H_4Ph) (left, for comparison) under UV irradiation.

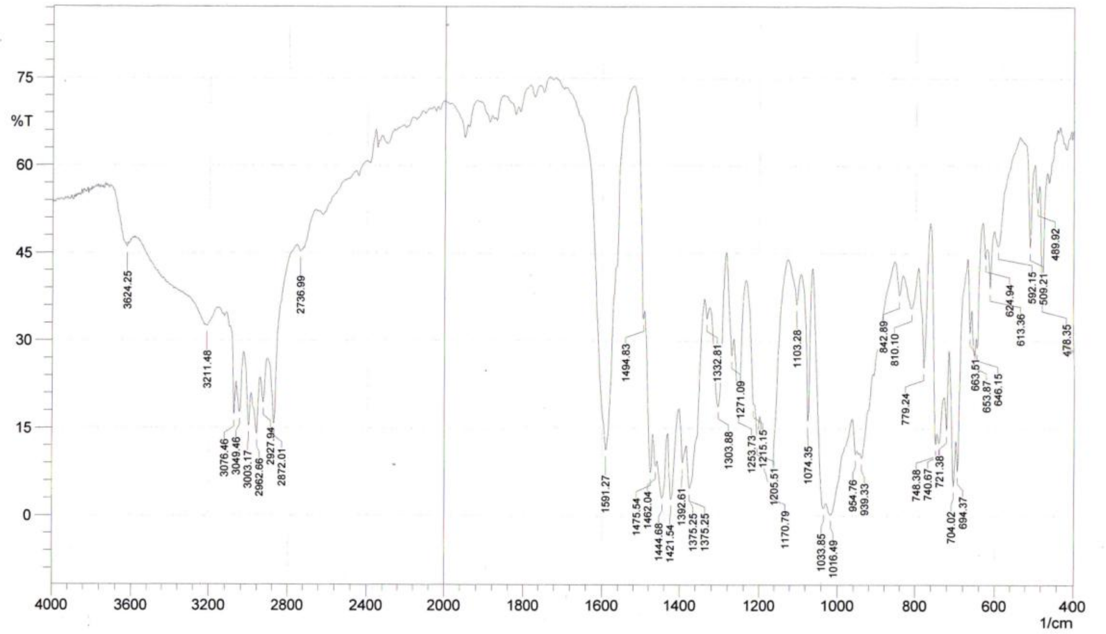

Supplementary Figure 3 | The solid-state IR spectrum of **4**.

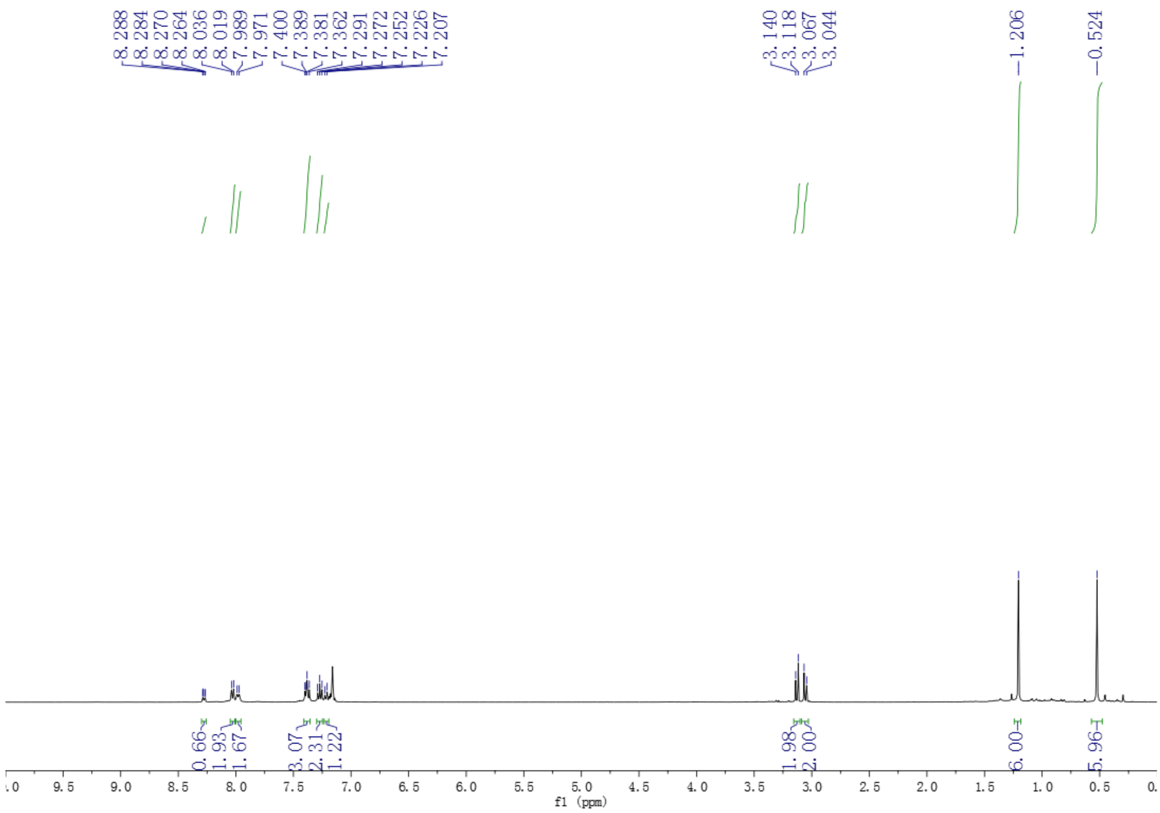

Supplementary Figure 4 | ^{1}H NMR spectrum of **3**.

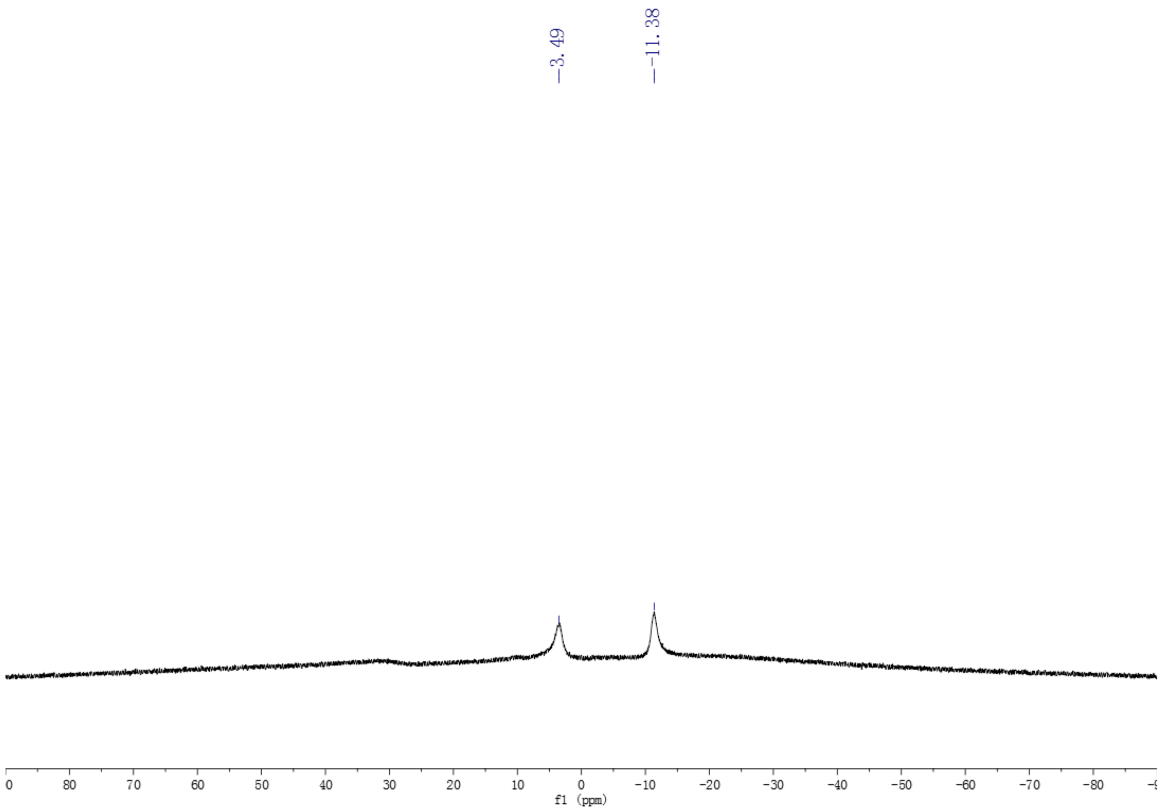

Supplementary Figure 5 | ^{11}B NMR spectrum of **3**.

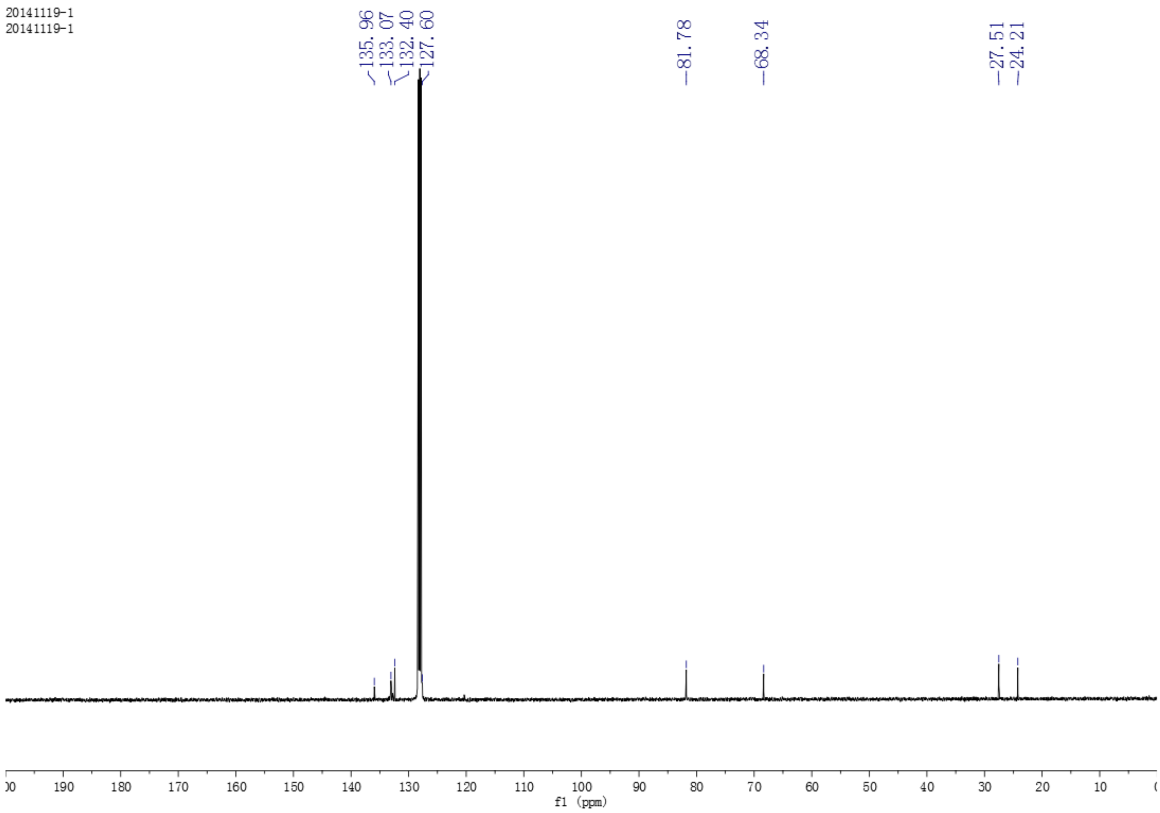

Supplementary Figure 6 | ^{13}C NMR spectrum of **3**.

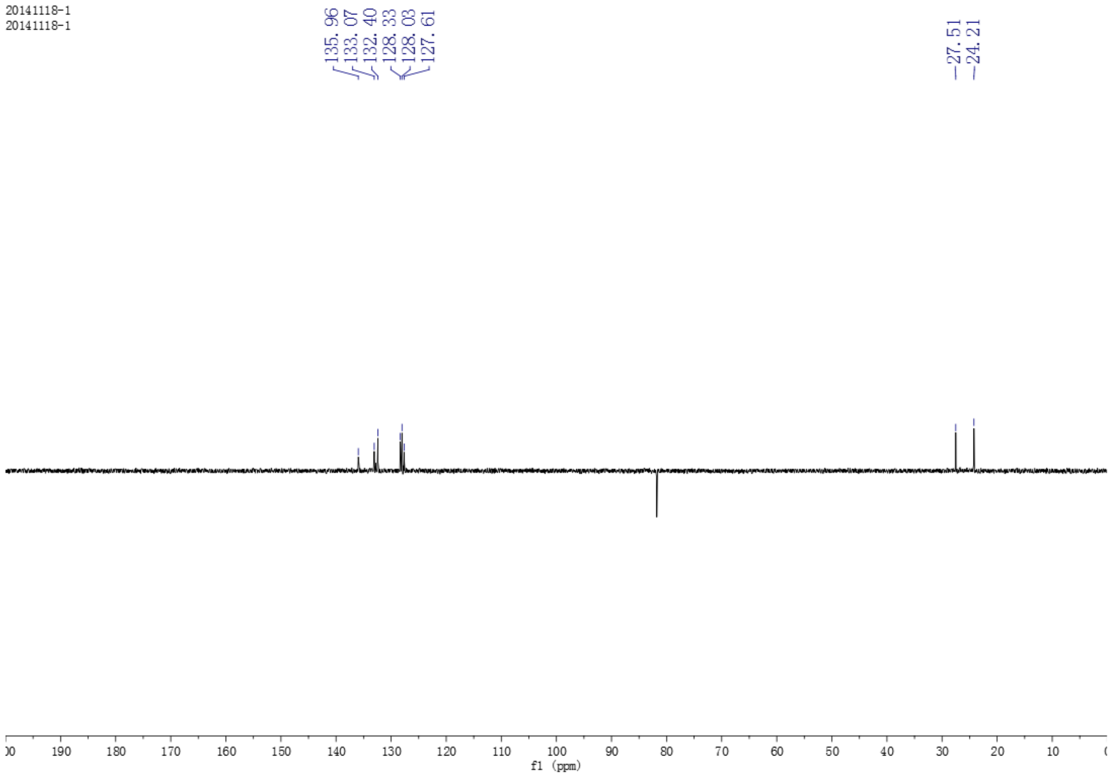

Supplementary Figure 7 | ^{13}C NMR (DEPT135) spectrum of **3**.

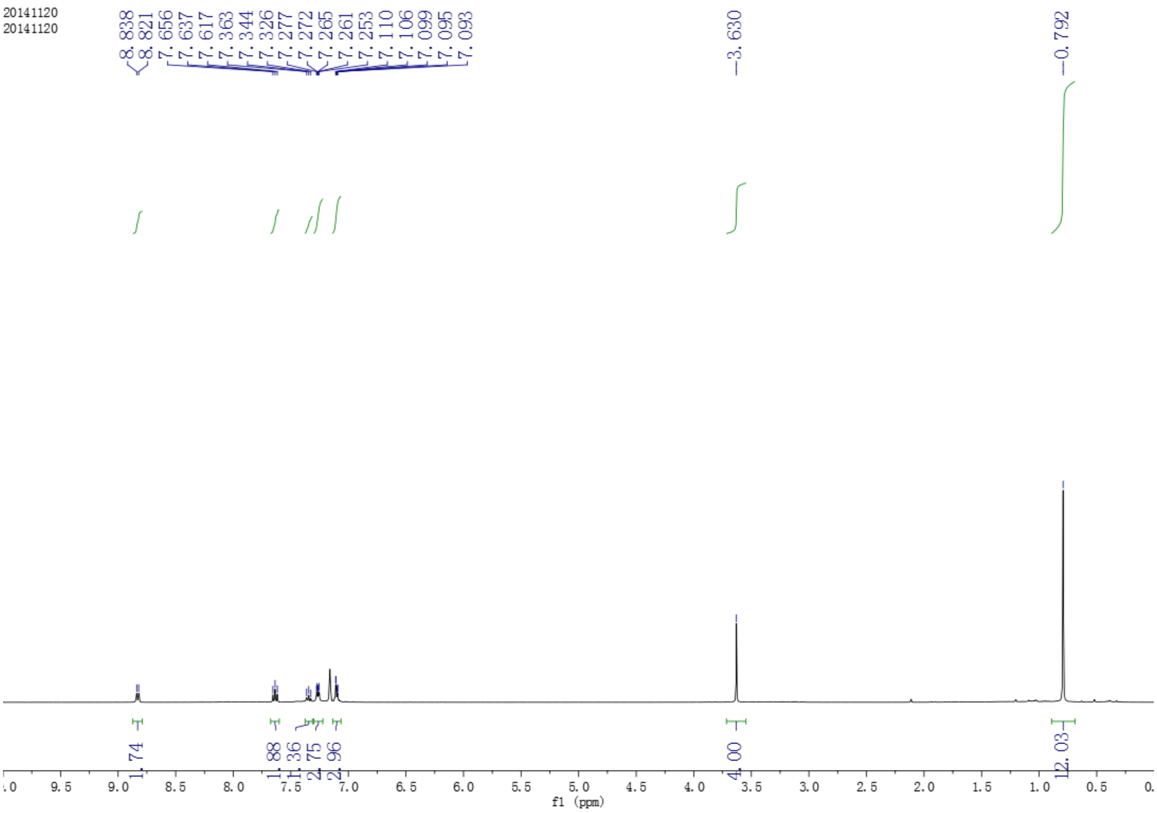

Supplementary Figure 8 | ^{1}H NMR spectrum of **4**.

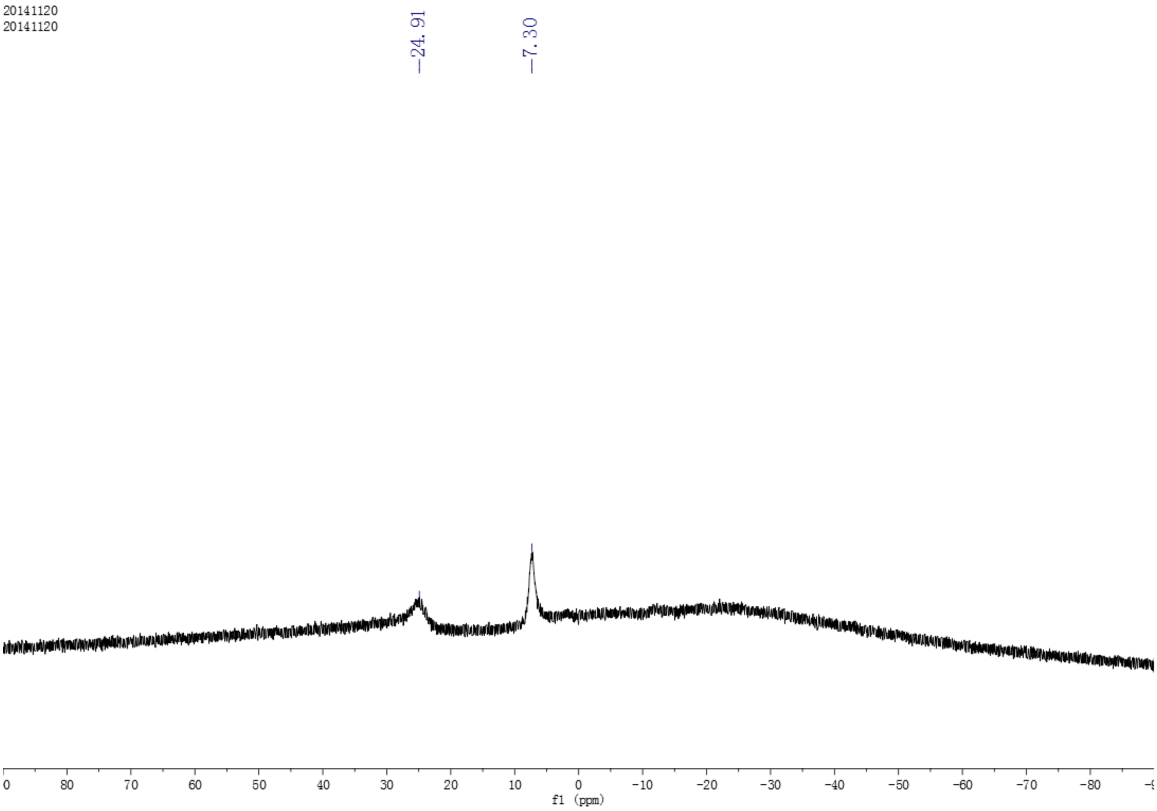

Supplementary Figure 9 | ^{11}B NMR spectrum of **4**.

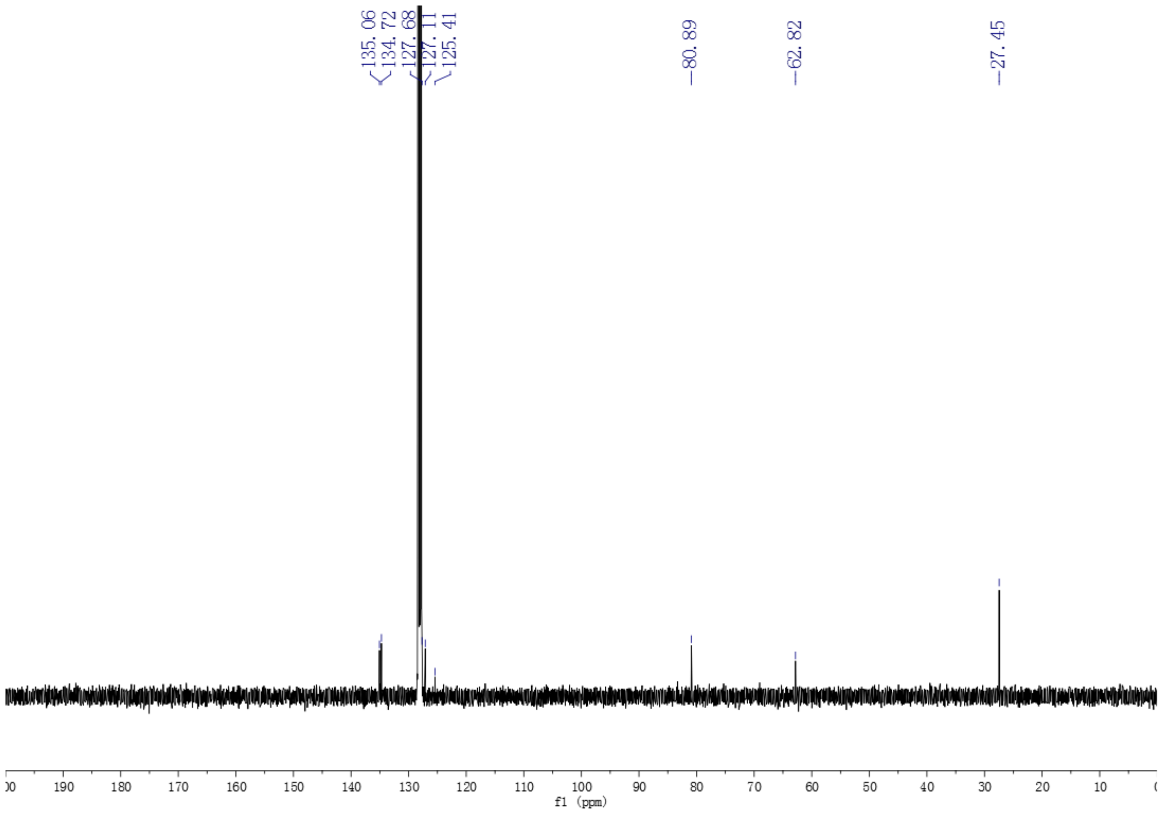

Supplementary Figure 10 | ^{13}C NMR spectrum of **4**.

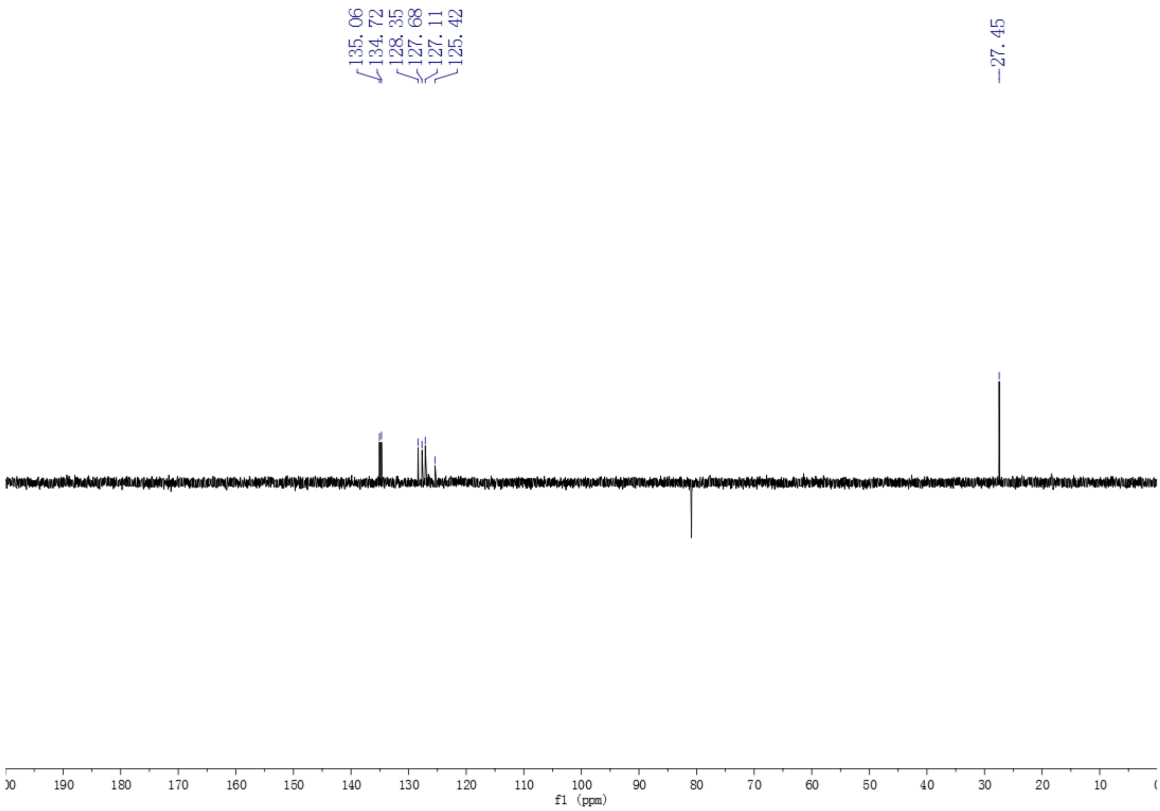

Supplementary Figure 11 | ^{13}C NMR (DEPT135) spectrum of **4**.

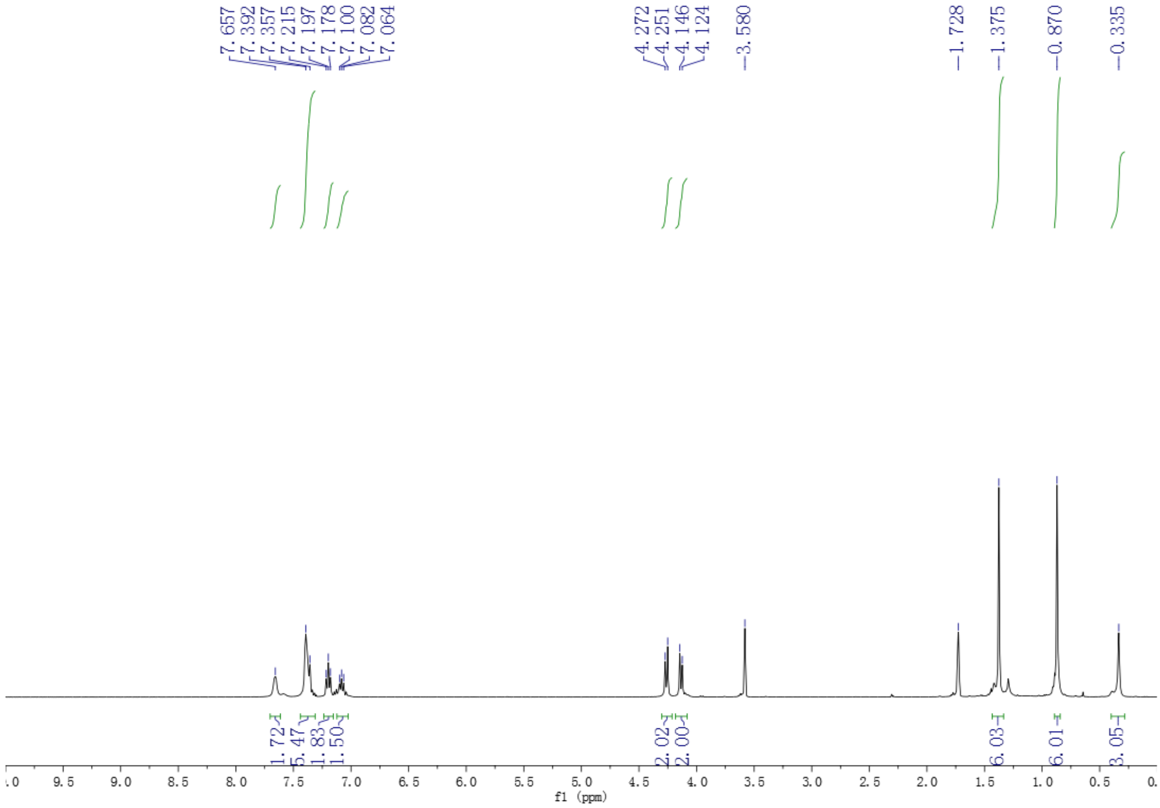

Supplementary Figure 12 | ^{1}H NMR spectrum of **5**.

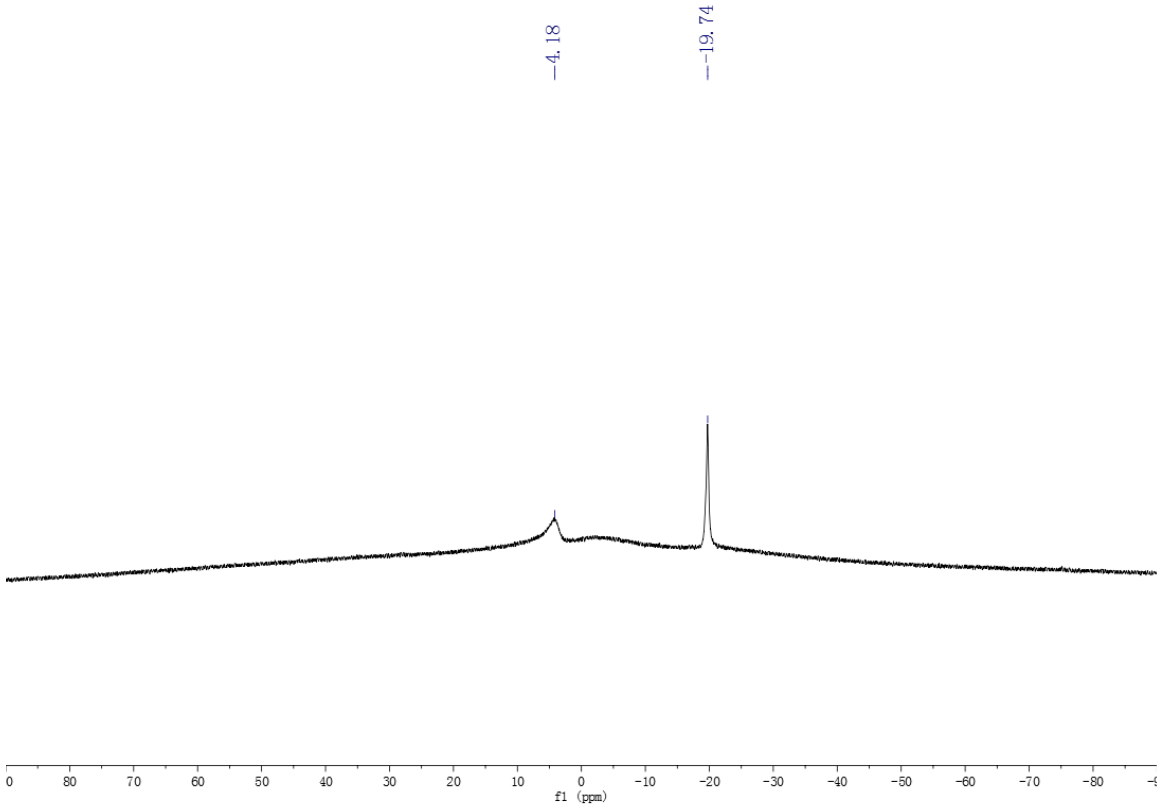

Supplementary Figure 13 | ^{11}B NMR spectrum of **5**.

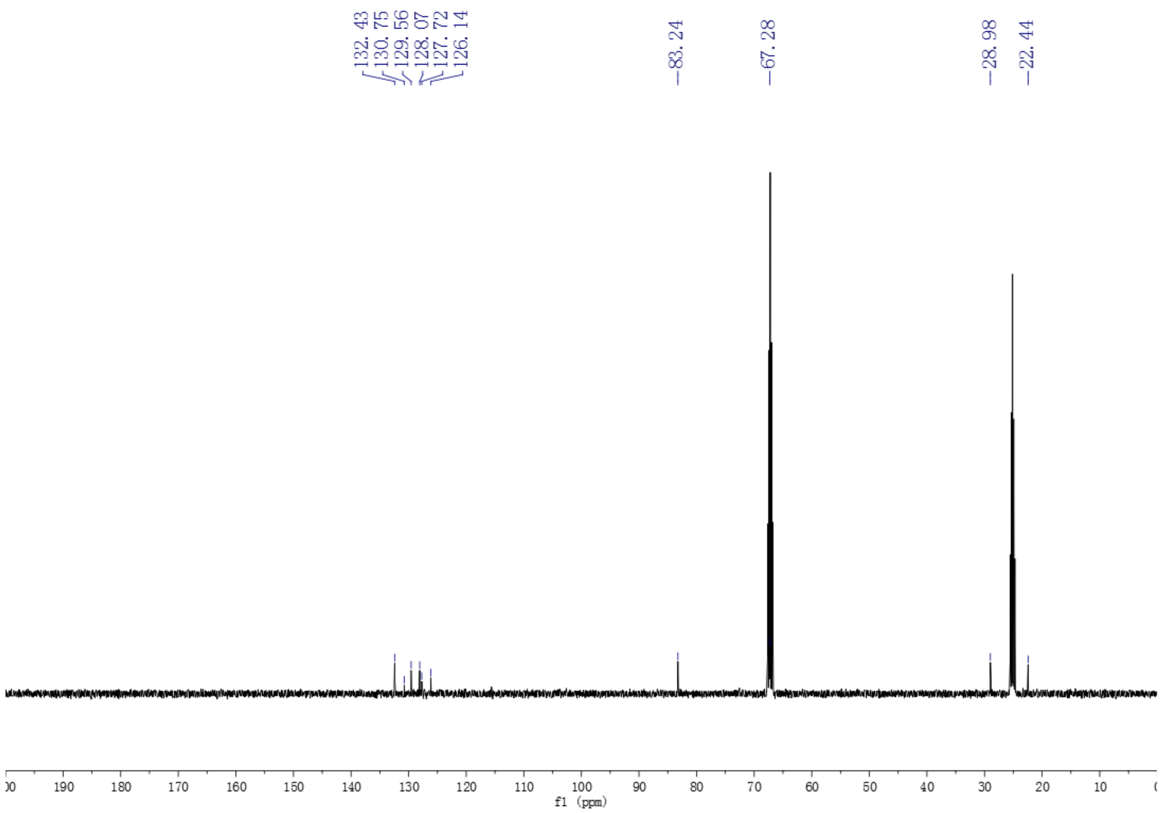

Supplementary Figure 14 | ^{13}C NMR spectrum of **5**.

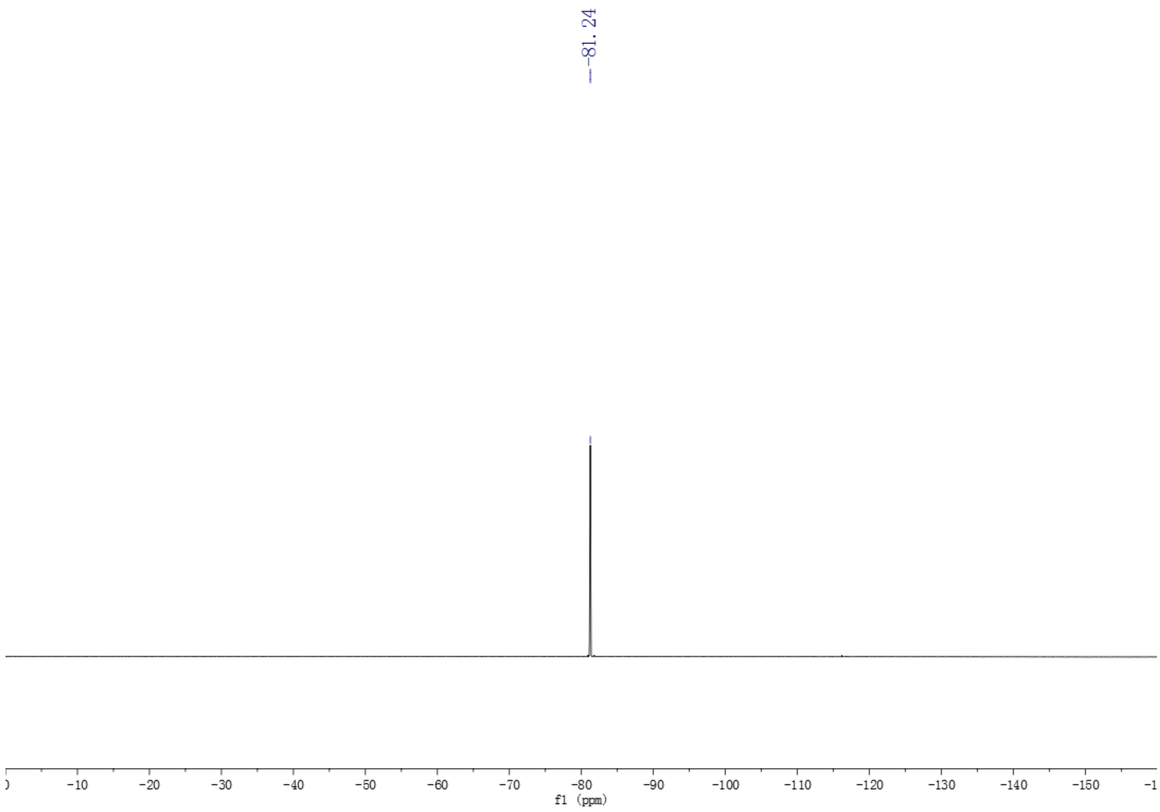

Supplementary Figure 15 | ^{19}F NMR spectrum of **5**.

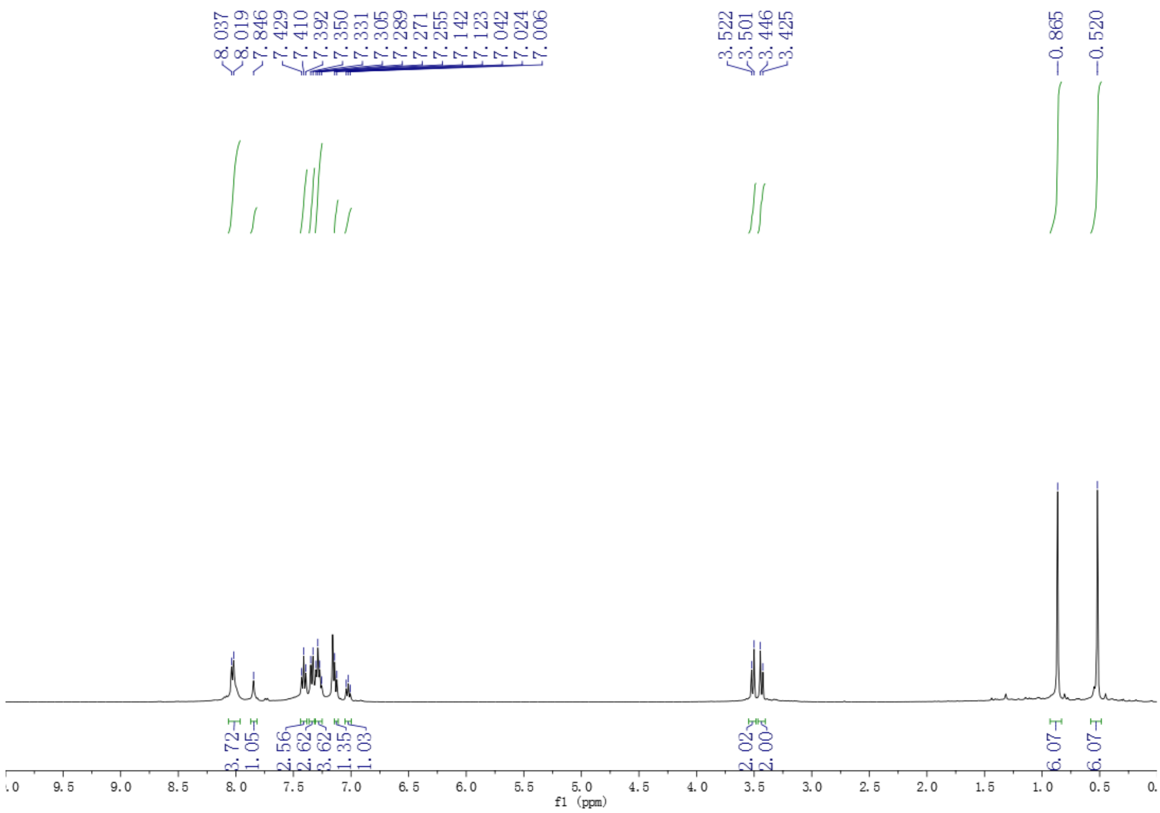

Supplementary Figure 16 | ^{1}H NMR spectrum of **6**.

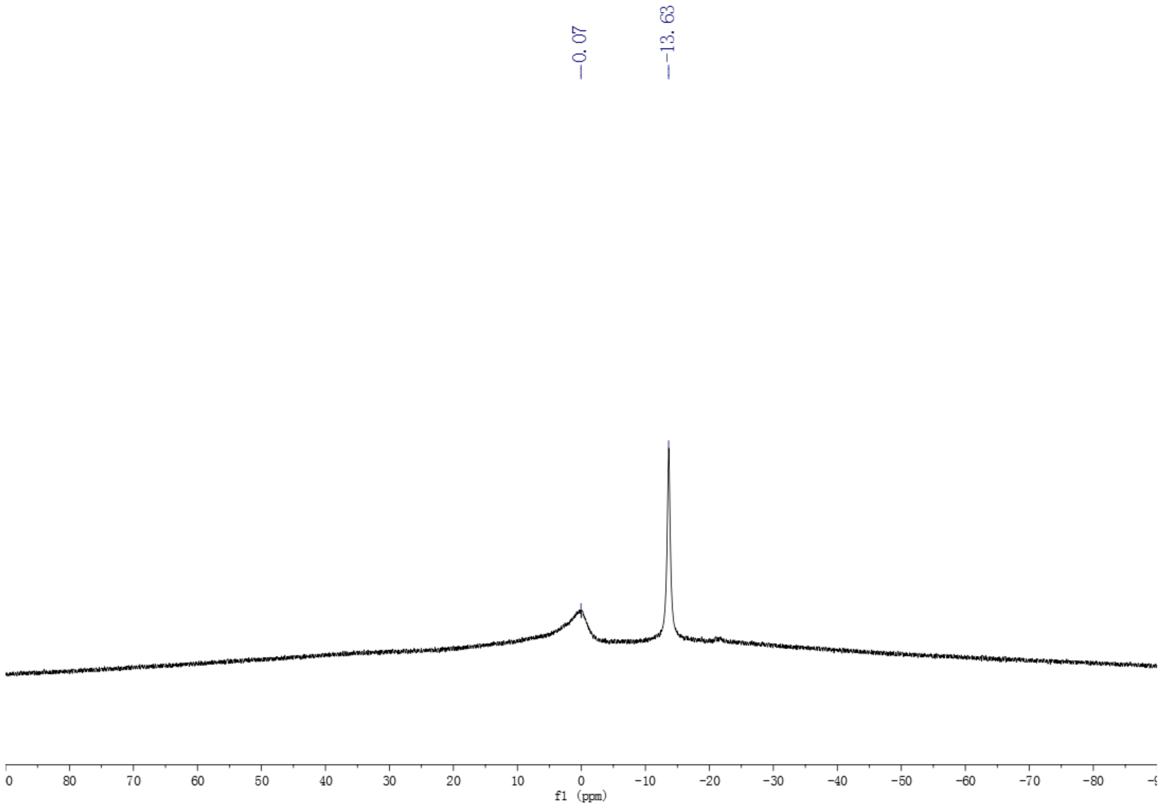

Supplementary Figure 17 | ^{11}B NMR spectrum of **6**.

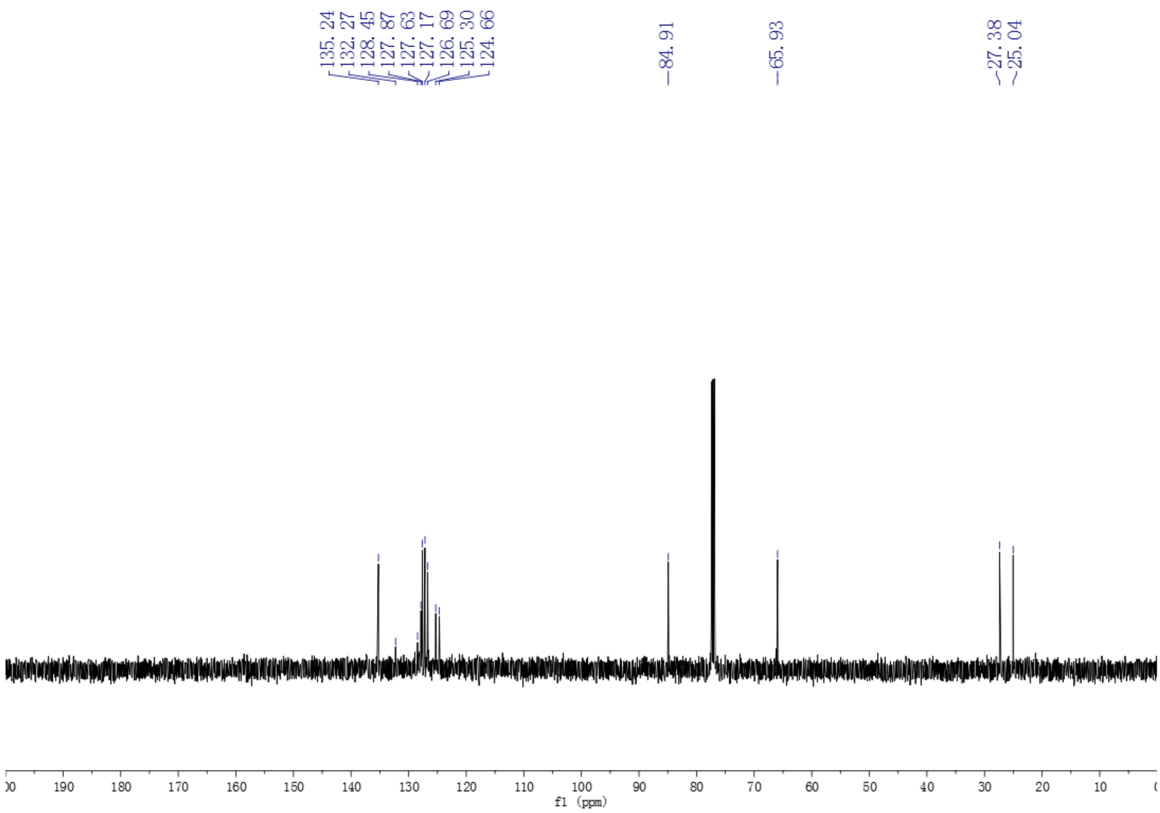

Supplementary Figure 18 | ^{11}C NMR spectrum of **6**.

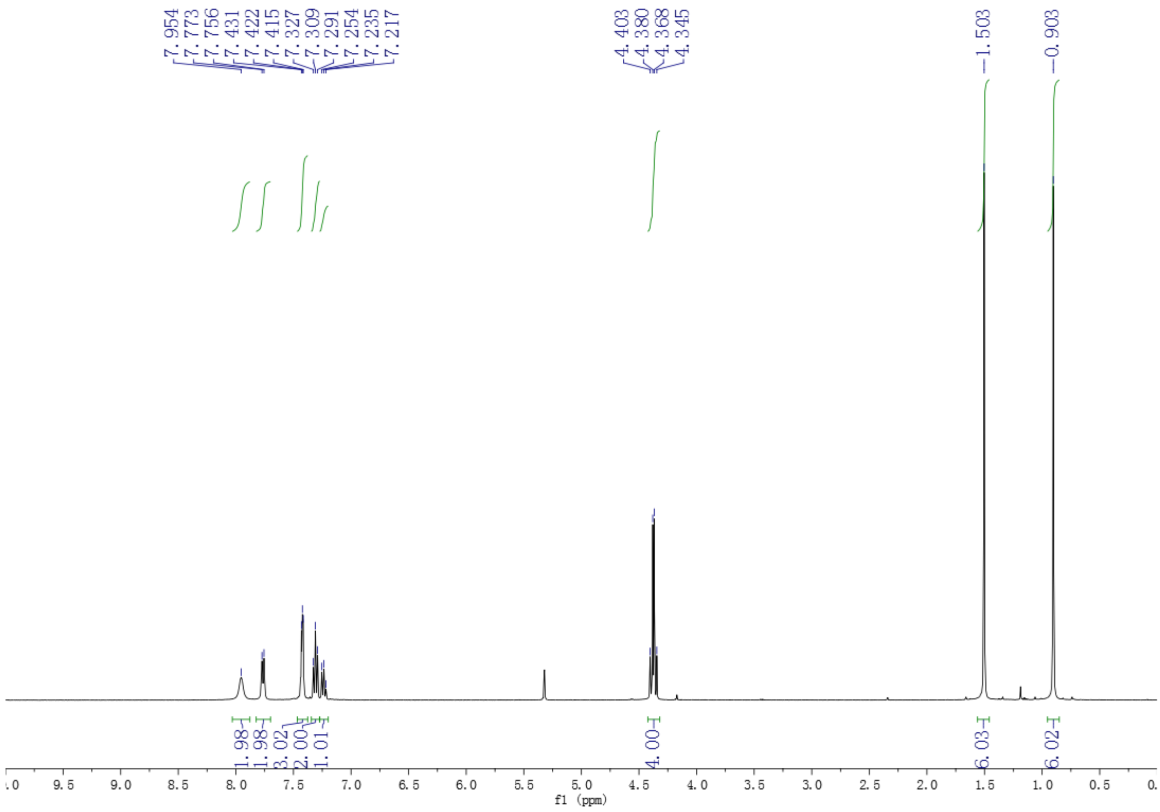

Supplementary Figure 19 | ^{1}H NMR spectrum of **7**.

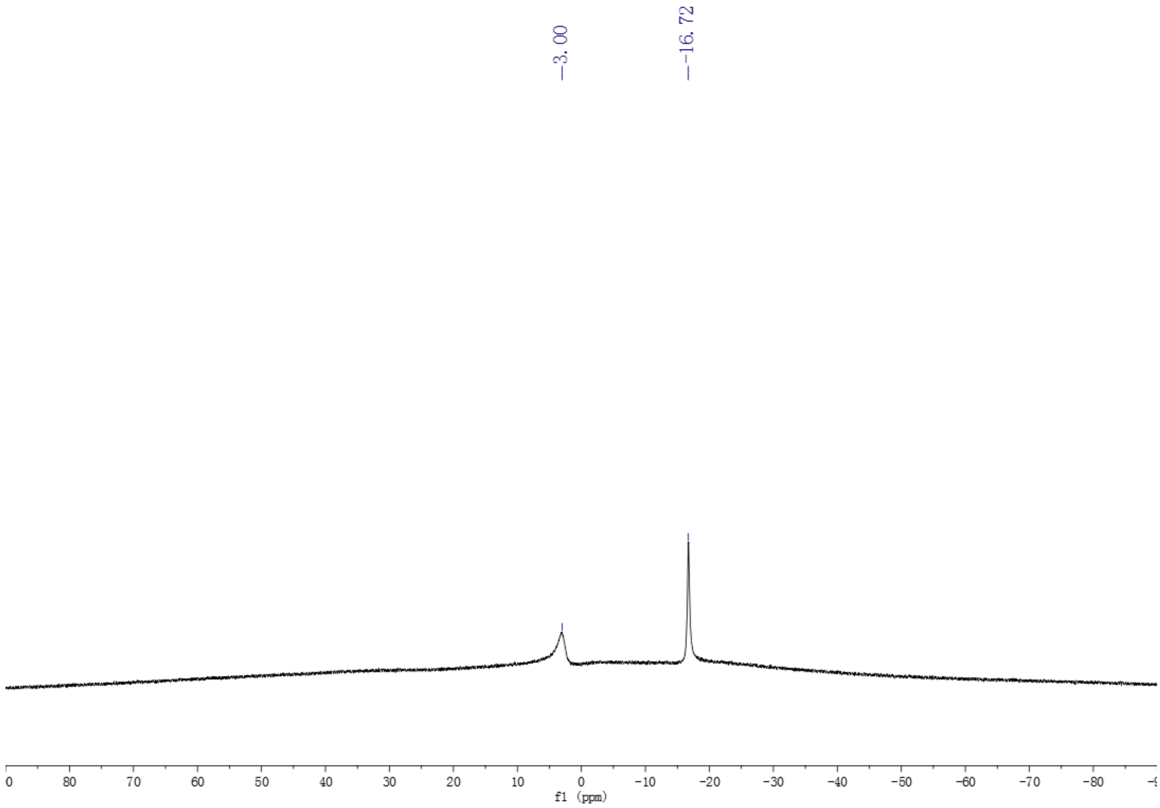

Supplementary Figure 20 | ^{11}B NMR spectrum of **7**.

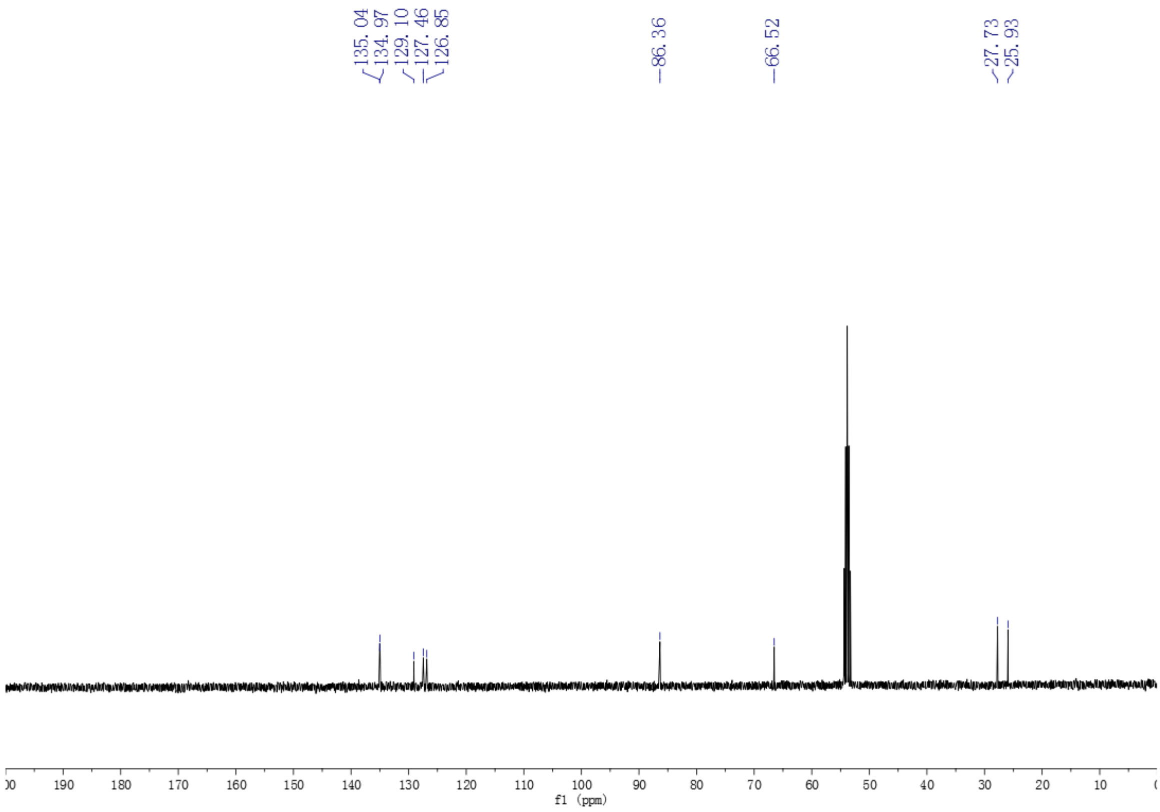

Supplementary Figure 21 | ^{13}C NMR spectrum of **7**.

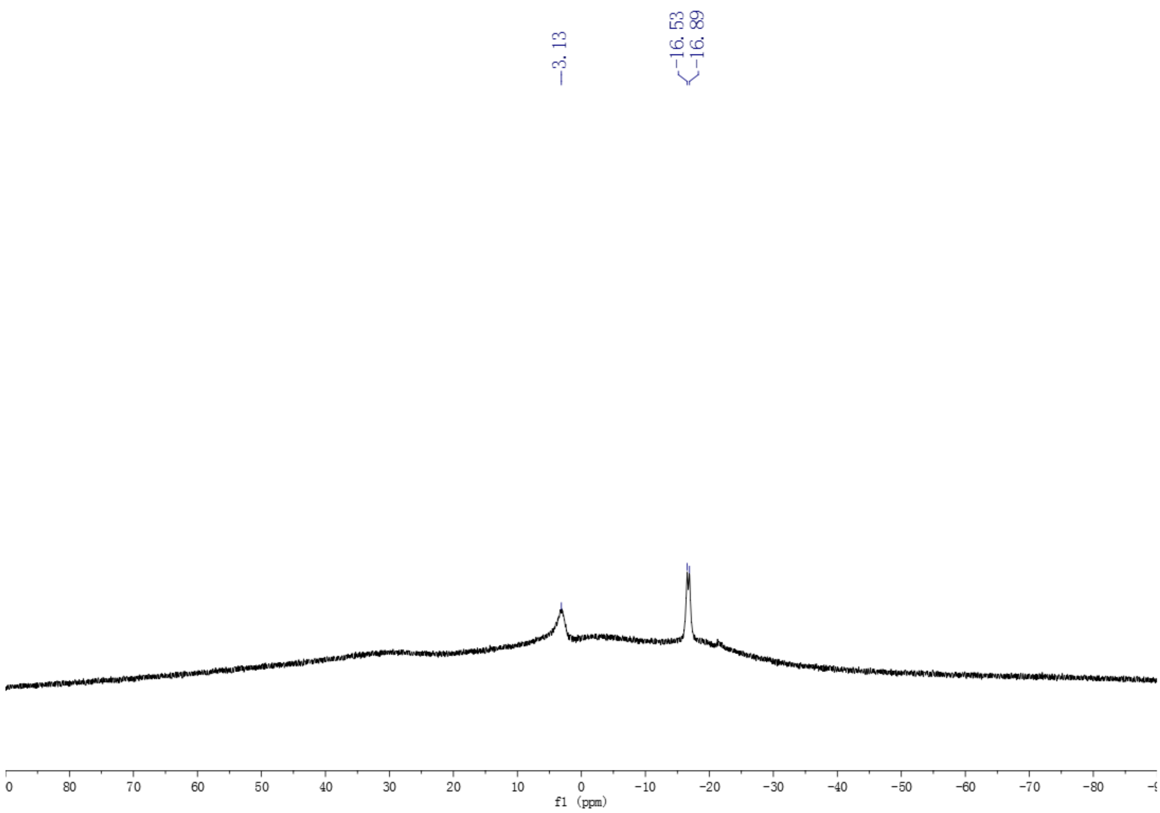

Supplementary Figure 22 | ^{11}B NMR spectrum of **7-^{13}C**.

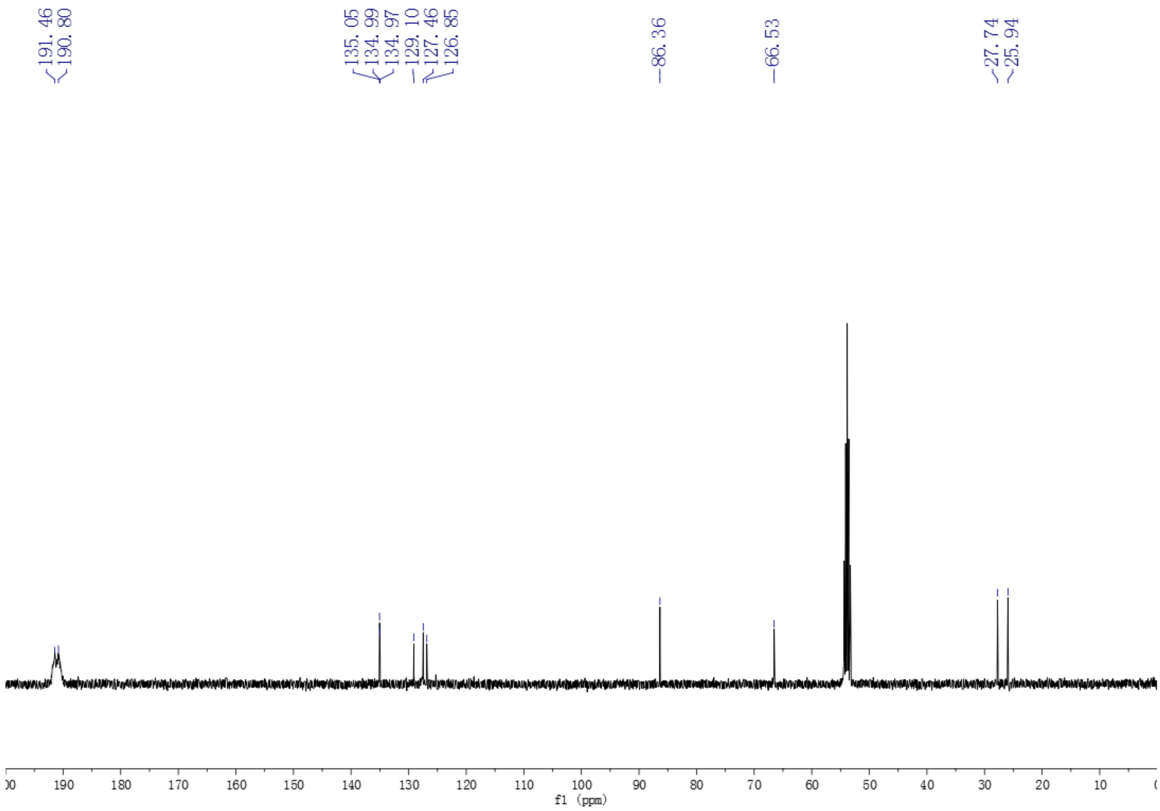

Supplementary Figure 23 | ^{13}C NMR spectrum of **7-^{13}C**.

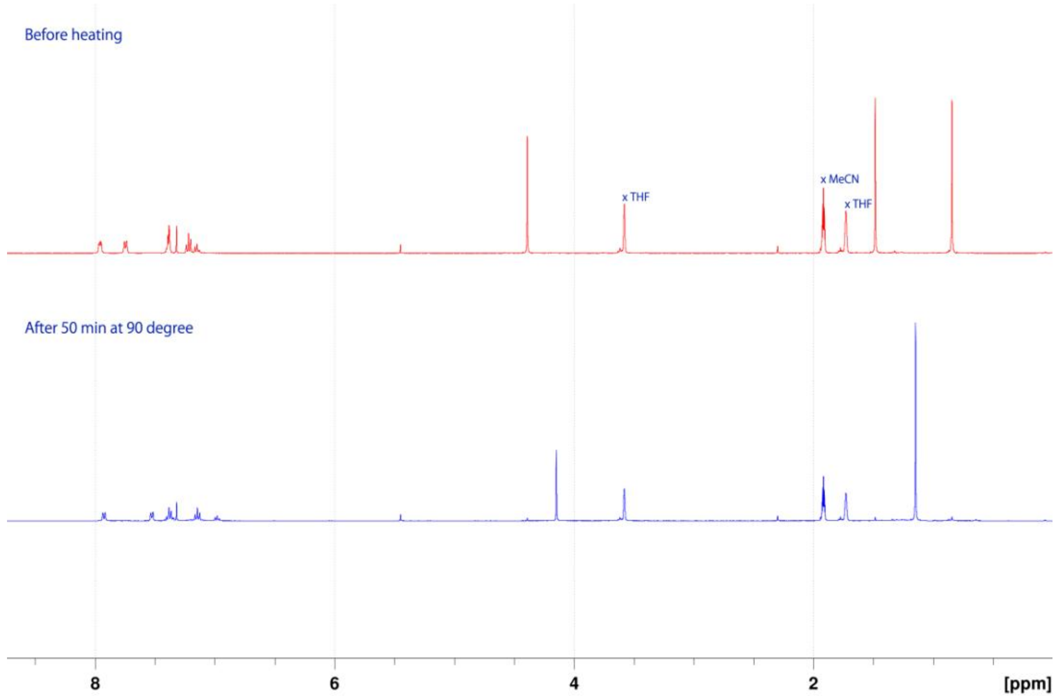

Supplementary Figure 24 | ^{1}H NMR spectrum showing the regeneration of **4** from **7**.

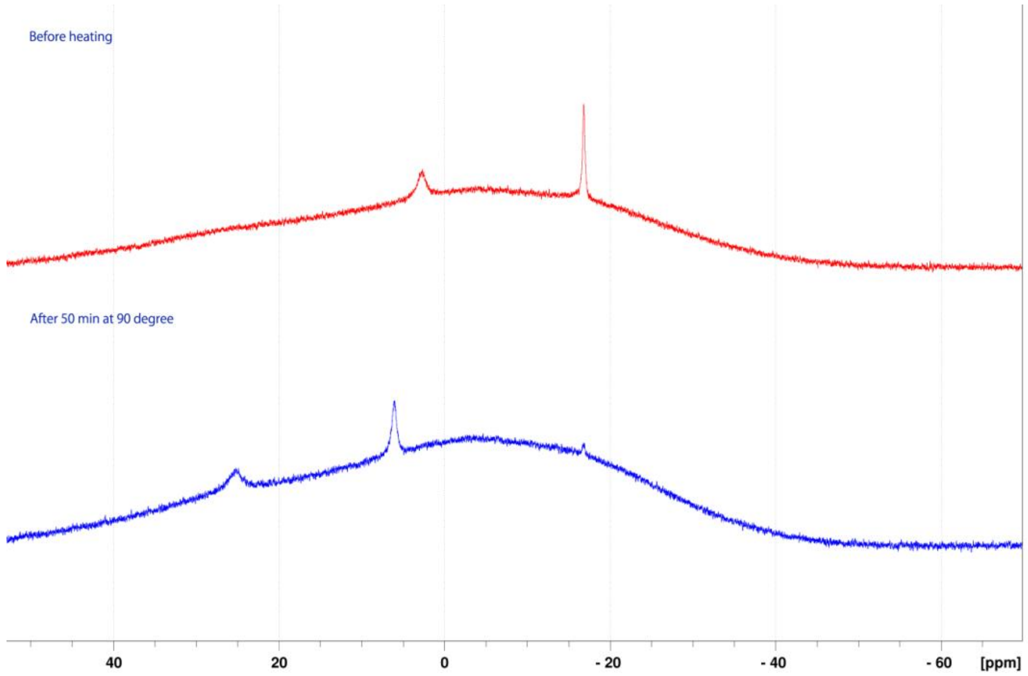

Supplementary Figure 25 | ^{11}B NMR spectrum showing the regeneration of **4** from **7**.

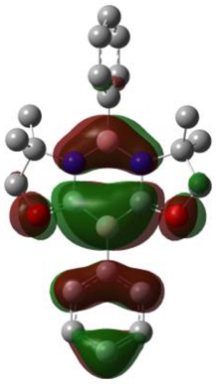

HOMO (– 4.5305)

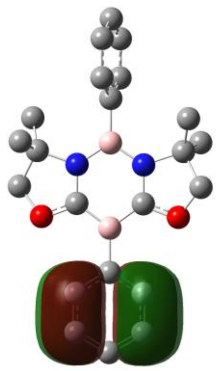

HOMO–1 (– 6.0774)

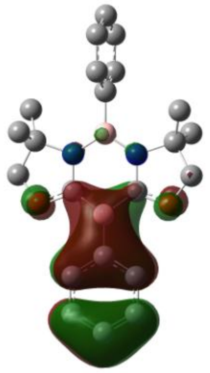

HOMO–2 (– 6.3471)

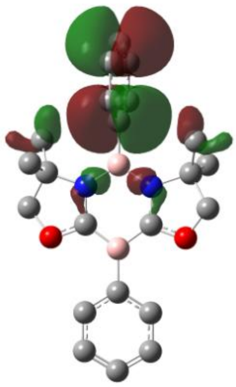

HOMO–3 (– 7.2475)

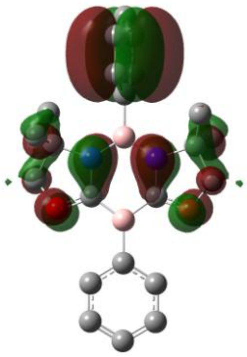

HOMO–4 (– 7.3273)

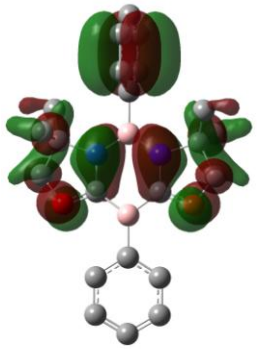

HOMO–5 (– 7.4367)

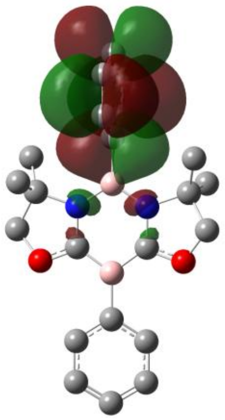

LUMO (– 0.8993)

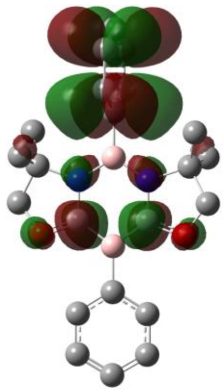

LUMO+1 (– 0.8362)

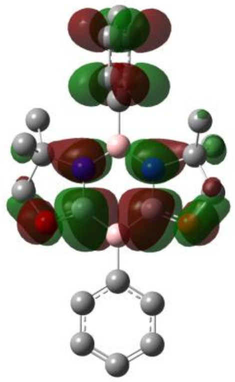

LUMO+2 (– 0.5714)

Supplementary Figure 26 | Plots of the frontier orbitals of **4**. The eigenvalues (eV) are provided in the parentheses**.**

Supplementary Tables

Supplementary Table 1. Summary of Data Collection and Structure Refinement.

	**$3{\cdot}(C_6H_6)_{0.5}$**	**4**	**$[5]_2{\cdot}(C_6H_5F)_5$**
Formula	C25H29B2Cl2N2O2	C22H26B2N2O2	C78H83B4F11N4O10S2
Fw	482.02	372.07	1552.84
cryst syst	triclinic	orthorhombic	triclinic
space group	*P-1*	*P b c n*	*P -1*
Size (mm^3)	0.140 x 0.160 x 0.240	0.040 x 0.400 x 0.420	0.060 x 0.100 x 0.420
T, K	296(2)	103(2)	103(2)
a, Å	9.205(3)	9.3775(15)	10.8994(10)
b, Å	10.740(3)	22.196(4)	13.3303(14)
c, Å	13.922(4)	9.4320(15)	14.0182(13)
α, deg	82.772(9)	90	98.334(4)
β, deg	76.229(9)	90	90.656(4)
γ, deg	74.293(9)	90	109.608(4)
V, A^3	1284.2(6)	1963.2(5)	1894.4(3)
Z	2	4	1
d_{calcd} $g{\cdot}cm^{-3}$	1.247	1.259	1.361
μ, mm^{-1}	0.277	0.079	0.159
Refl collected	15505	2451	27307
T_{min}/T_{max}	0.9360/0.9620	0.9680/0.9970	0.9360/0.9910
N_{measd}	4454	2451	9409
[R_{int}]	0.0528	0.0939	0.1022
R [I>2sigma(I)]	0.0629	0.0672	0.0790
R_w[I>2sigma(I)]	0.2094	0.2035	0.2145
GOF	1.032	0.848	1.017
Largest diff peak/hole[$e{\cdot}Å^{-3}$]	0.270/-0.270	0.323/-0.237	1.084/-0.466

Supplementary Table 2. Summary of Data Collection and Structure Refinement.

	**6·(thf)$_{0.75}$·(C$_6$H$_6$)$_{0.25}$**	**7·(CH$_2$Cl$_2$)$_{0.5}$**
Formula	C34.50H39.50B2N2O2.75	C23.50H27B2ClN2O4
Fw	547.80	458.54
cryst syst	monoclinic	monoclinic
space group	*P 1 21/n 1*	*P 1 21/c 1*
Size (mm^3)	0.040 x 0.320 x 0.380	0.020 x 0.100 x 0.280
T, K	103(2)	103(2)
a, Å	11.3572(9)	13.0170(16)
b, Å	9.8183(8)	6.3932(8)
c, Å	27.031(2)	27.756(3)
α, deg	90	90
β, deg	96.547(4)	99.795(4)
γ, deg	90	90
V, A^3	2994.5(4)	2276.2(5)
Z	4	4
d_{calcd}g·cm^{-3}	1.215	1.338
μ, mm^{-1}	0.075	0.202
Refl collected	25342	17919
T_{min}/ T_{max}	0.9720/0.9970	0.9460/0.9960
N $_{measd}$	5884	4020
[R$_{int}$]	0.0863	0.1490
R [I>2sigma(I)]	0.0689	0.0730
R_w[I>2sigma(I)]	0.2237	0.2232
GOF	1.007	1.053
Largest diff peak/hole[e·Å^{-3}]	0.553 /-0.441	0.424/-0.480

Supplementary Table 3. Optimized structures of **4**, **4'**, **4'+2H**, **C_6H_6**, **C_6H_8**, **1,2-azaborine**, **1,3-azaborine**, **borazine** (atom, x-, y-, z-positions in Å).

4:

Zero-point correction=	0.448145 (Hartree/Particle)
Thermal correction to Energy=	0.473068
Thermal correction to Enthalpy=	0.474012
Thermal correction to Gibbs Free Energy=	0.393492
Sum of electronic and zero-point Energies=	-1163.607923
Sum of electronic and thermal Energies=	-1163.583000
Sum of electronic and thermal Enthalpies=	-1163.582056
Sum of electronic and thermal Free Energies=	-1163.662576

B	1.878351	-0.000009	-0.00012
C	3.453532	-0.00012	-0.00031
C	1.005141	-1.21025	-0.0883
C	1.005329	1.210243	0.0884
C	4.199442	1.157614	-0.30705
C	4.199408	-1.15792	0.306222
N	-0.38028	-1.21211	-0.09208
O	1.476988	-2.48525	-0.16397
N	-0.38015	1.212253	0.092443
O	1.477247	2.485274	0.164427
C	5.592996	1.160029	-0.3101
C	5.592966	-1.16046	0.308939
B	-1.15341	0.000177	0.00014
C	-0.866	-2.63868	-0.05693
C	0.404559	-3.34795	-0.54714
C	-0.86573	2.638872	0.058144
C	0.404991	3.34771	0.548543
C	6.300943	-0.00025	-0.00066
C	-2.73753	0.00018	-0.00017
C	-1.18865	-3.04012	1.391249
C	-2.03339	-2.94775	-0.99576
C	-1.18857	3.04129	-1.38969
C	-2.03295	2.947332	0.99745
C	-3.47157	-0.14923	1.188496
C	-3.47051	0.149423	-1.18954
C	-4.866	-0.15096	1.192961
C	-4.86494	0.150814	-1.1953

C	-5.56849	-0.00017	-0.0015
H	3.678785	2.076522	-0.54537
H	3.678741	-2.0768	0.544628
H	6.128073	2.072472	-0.55456
H	6.128019	-2.07296	0.553247
H	0.560622	-4.32297	-0.08576
H	0.409587	-3.45269	-1.63839
H	0.560917	4.323084	0.087864
H	0.41029	3.451657	1.63988
H	7.38585	-0.00029	-0.00078
H	-0.32661	-2.87411	2.041624
H	-1.45583	-4.10005	1.43571
H	-2.03177	-2.46419	1.774555
H	-2.13176	-4.03427	-1.08669
H	-2.97875	-2.55811	-0.6236
H	-1.85454	-2.54047	-1.99313
H	-0.32663	2.875653	-2.04029
H	-1.4557	4.101263	-1.43337
H	-2.03179	2.465669	-1.77324
H	-2.13094	4.033767	1.089757
H	-2.97846	2.558425	0.624881
H	-1.85411	2.538783	1.994312
H	-2.94701	-0.2549	2.132987
H	-2.9451	0.255189	-2.13354
H	-5.40325	-0.26686	2.128111
H	-5.40136	0.266598	-2.13094
H	-6.65282	-0.00033	-0.00199

4’:

Zero-point correction= 0.094862 (Hartree/Particle)
Thermal correction to Energy= 0.099773
Thermal correction to Enthalpy= 0.100717
Thermal correction to Gibbs Free Energy= 0.067007
Sum of electronic and zero-point Energies= -239.072496
Sum of electronic and thermal Energies= -239.067585
Sum of electronic and thermal Enthalpies= -239.066640
Sum of electronic and thermal Free Energies= -239.100350

C	1.250816	0.711817	-0.000071
C	-1.250816	0.711817	-0.000071
B	0.000000	1.531075	0.000246
B	0.000000	-1.417332	0.000056
N	-1.205184	-0.65517	-0.000088
N	1.205184	-0.655171	-0.000086
H	2.260961	1.115716	0.000431
H	-2.26096	1.115717	0.000433
H	-0.000006	2.722544	-0.000622
H	-0.000004	-2.602941	0.000401
H	-2.090784	-1.144584	-0.000039
H	2.090784	-1.144584	-0.000044

4’ + 2H:

Zero-point correction= 0.114282 (Hartree/Particle)
Thermal correction to Energy= 0.120529
Thermal correction to Enthalpy= 0.121473
Thermal correction to Gibbs Free Energy= 0.084952
Sum of electronic and zero-point Energies= -240.261436
Sum of electronic and thermal Energies= -240.255189
Sum of electronic and thermal Enthalpies= -240.254245
Sum of electronic and thermal Free Energies= -240.290765

N	-1.233565	-0.744901	0.00035
C	-1.363227	0.709251	-0.000096
B	-0.000065	1.470218	0.000036
C	1.363145	0.709354	0.000102
N	1.23365	-0.744807	-0.000371
B	0.000067	-1.441596	-0.000021
H	-2.096481	-1.2644	-0.000662
H	-1.942775	1.068251	0.868784
H	-1.942673	1.067677	-0.869292
H	-0.000086	2.665297	-0.000096

H	1.942544	1.067845	0.869307
H	1.942634	1.068446	-0.868783
H	2.096618	-1.264219	0.00066
H	0.000109	-2.635675	0.000111

C_6H_6:

Zero-point correction= 0.100180 (Hartree/Particle)
Thermal correction to Energy= 0.104577
Thermal correction to Enthalpy= 0.105521
Thermal correction to Gibbs Free Energy= 0.072716
Sum of electronic and zero-point Energies= -232.211092
Sum of electronic and thermal Energies= -232.206694
Sum of electronic and thermal Enthalpies= -232.205750
Sum of electronic and thermal Free Energies= -232.238555

C	0.994697	-0.977063	-0.000002
C	-0.348965	-1.349818	-0.000062
C	-1.343571	-0.372874	0.000056
C	-0.994612	0.977153	-0.000006
C	0.348848	1.349846	-0.000057
C	1.343604	0.372759	0.000051
H	1.768042	-1.737054	0.000063
H	-0.62012	-2.399665	-0.00008
H	-2.388372	-0.662728	0.00012
H	-1.768146	1.736942	-0.000021
H	0.620252	2.399624	0.00000
H	2.38834	0.66287	0.000039

C_6H_8:

Zero-point correction= 0.121816 (Hartree/Particle)
Thermal correction to Energy= 0.126997
Thermal correction to Enthalpy= 0.127941
Thermal correction to Gibbs Free Energy= 0.093587
Sum of electronic and zero-point Energies= -233.362082
Sum of electronic and thermal Energies= -233.356902
Sum of electronic and thermal Enthalpies= -233.355958
Sum of electronic and thermal Free Energies= -233.390312

C	-1.192598	0.728736	0.24464
C	-1.192597	-0.728736	-0.24464
C	0.112453	-1.422939	0.064447
C	1.257155	-0.724991	0.107569
C	1.257154	0.724992	-0.107569

C	0.112453	1.422939	-0.064447
H	-2.03517	1.271841	-0.19234
H	-1.346784	0.752278	1.335084
H	-2.035169	-1.271842	0.19234
H	-1.346783	-0.752279	-1.335084
H	0.115964	-2.50138	0.186412
H	2.20393	-1.225848	0.28141
H	2.203929	1.225849	-0.28141
H	0.115963	2.50138	-0.186412

1,2-azaborine:

Zero-point correction= 0.097528 (Hartree/Particle)
Thermal correction to Energy= 0.102216
Thermal correction to Enthalpy= 0.103160
Thermal correction to Gibbs Free Energy= 0.069846
Sum of electronic and zero-point Energies= -235.648358
Sum of electronic and thermal Energies= -235.643671
Sum of electronic and thermal Enthalpies= -235.642726
Sum of electronic and thermal Free Energies= -235.676040

C	-1.328928	-0.48383	-0.000072
C	-1.180707	0.883521	0.00002
C	1.061458	-0.852305	0.000026
C	-0.207243	-1.359651	-0.000009
N	1.273707	0.497577	-0.000021
B	0.213509	1.468541	-0.000005
H	-2.318576	-0.935162	0.000105
H	-2.077607	1.495617	0.000115
H	1.936754	-1.491455	0.000084
H	-0.346092	-2.43321	0.00007
H	2.240487	0.791285	-0.00004
H	0.51406	2.620767	0.000044

1,3-azaborine:

Zero-point correction= 0.097135 (Hartree/Particle)
Thermal correction to Energy= 0.101839
Thermal correction to Enthalpy= 0.102784
Thermal correction to Gibbs Free Energy= 0.069445
Sum of electronic and zero-point Energies= -235.601676
Sum of electronic and thermal Energies= -235.596972
Sum of electronic and thermal Enthalpies= -235.596028
Sum of electronic and thermal Free Energies= -235.629366

C	-0.246756	-1.315986	0.00000
C	1.076213	-0.89398	0.00000
C	1.405634	0.460454	0.00000
C	-1.066875	0.942928	0.00000
N	-1.244983	-0.402301	0.00000
B	0.324148	1.511861	0.00000
H	-0.546254	-2.35455	0.00000
H	1.839382	-1.666786	0.00000
H	2.466998	0.699196	0.00000
H	-2.002073	1.493835	0.00000
H	-2.190799	-0.764248	0.00000
H	0.517587	2.688857	0.00000

Borazine:

Zero-point correction= 0.093177 (Hartree/Particle)
Thermal correction to Energy= 0.098386
Thermal correction to Enthalpy= 0.099330
Thermal correction to Gibbs Free Energy= 0.065055
Sum of electronic and zero-point Energies= -242.655338
Sum of electronic and thermal Energies= -242.650129
Sum of electronic and thermal Enthalpies= -242.649185
Sum of electronic and thermal Free Energies= -242.683460

N	0.591717	-1.278825	-0.000011
N	-1.403508	0.127029	0.000018
B	-0.835878	-1.186124	-0.00008
B	1.445222	-0.130819	0.000023
B	-0.609298	1.316899	-0.000042
N	0.81175	1.151837	0.000001
H	1.015327	-2.194365	0.000066
H	-2.408193	0.217949	0.000129
H	-1.522841	-2.161035	0.00015
H	2.632996	-0.23838	0.000029
H	-1.110078	2.399316	0.000045
H	1.392846	1.976444	0.000022

Supplementary Table 4. Raw energy data for RSE calculation.

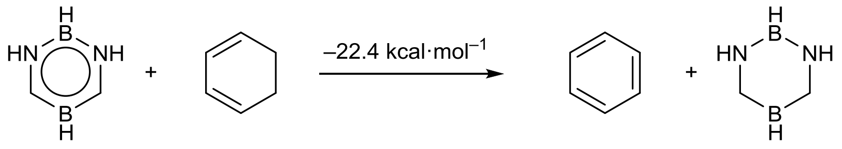

	total energy (au)	ZPE	H_{corr}	G_{corr}	E + ZPE	E + H_{corr}	E + G_{corr}
**4'**	-239.1673577	0.09486	0.10072	0.06701	-239.072496	-239.066641	-239.100351
**4'+2H**	-240.3757176	0.11428	0.12147	0.08495	-240.261436	-240.254245	-240.290766
**C_6H_6**	-232.3112711	0.10018	0.10552	0.07272	-232.211091	-232.20575	-232.238555
**C_6H_8**	-233.4838985	0.12182	0.12794	0.09359	-233.362082	-233.355957	-233.390311
**[(4'+2H) + C_6H_6] - (4' + C_6H_8) [au]**	-0.03573264				-0.03794864	-0.03739664	-0.03865864
**[kcal mol^{-1}]**	-22.42257285				-23.813134	-23.4667487	-24.2586658

H_{corr} = Enthalpy correction. G_{corr} = Free energy correction.

Supplementary Table 5. The NPA charges of **4**.

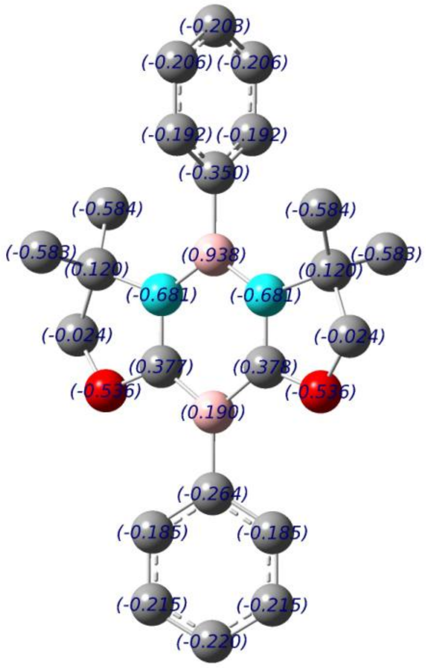

Atom	No	Natural Charge	Core	Valence	Rydberg	Total
B	1	0.18980	1.99864	2.79544	0.01613	4.81020
C	2	-0.26443	1.99898	4.24683	0.01862	6.26443
C	3	0.37751	1.99875	3.59113	0.03261	5.62249
C	4	0.37739	1.99875	3.59126	0.03261	5.62261
C	5	-0.18481	1.99911	4.16930	0.01640	6.18481
C	6	-0.18482	1.99911	4.16931	0.01640	6.18482
N	7	-0.68128	1.99918	5.66329	0.01881	7.68128
O	8	-0.53626	1.99973	6.51920	0.01733	8.53626
N	9	-0.68131	1.99918	5.66332	0.01881	7.68131
O	10	-0.53629	1.99973	6.51923	0.01733	8.53629
H	11	0.20656	0.00000	0.79159	0.00185	0.79344
C	12	-0.21458	1.99915	4.19697	0.01847	6.21458
H	13	0.20656	0.00000	0.79159	0.00185	0.79344
C	14	-0.21458	1.99915	4.19697	0.01847	6.21458
B	15	0.93847	1.99884	2.04402	0.01867	4.06153
C	16	0.12014	1.99917	3.86084	0.01986	5.87986
C	17	-0.02447	1.99904	4.00374	0.02169	6.02447
C	18	0.12014	1.99917	3.86083	0.01986	5.87986
C	19	-0.02447	1.99904	4.00373	0.02169	6.02447
H	20	0.19619	0.00000	0.80220	0.00161	0.80381

C	21	-0.22030	1.99915	4.20190	0.01925	6.22030
H	22	0.19619	0.00000	0.80220	0.00161	0.80381
C	23	-0.35035	1.99897	4.32572	0.02567	6.35035
C	24	-0.58312	1.99926	4.57296	0.01090	6.58312
C	25	-0.58400	1.99925	4.57254	0.01220	6.58400
H	26	0.19563	0.00000	0.80291	0.00146	0.80437
H	27	0.17890	0.00000	0.81915	0.00195	0.82110
C	28	-0.58313	1.99926	4.57297	0.01090	6.58313
C	29	-0.58400	1.99925	4.57254	0.01220	6.58400
H	30	0.19562	0.00000	0.80291	0.00146	0.80438
H	31	0.17889	0.00000	0.81916	0.00195	0.82111
H	32	0.19730	0.00000	0.80119	0.00151	0.80270
C	33	-0.19224	1.99908	4.17599	0.01717	6.19224
C	34	-0.19223	1.99908	4.17598	0.01717	6.19223
H	35	0.20825	0.00000	0.79055	0.00120	0.79175
H	36	0.22337	0.00000	0.77514	0.00149	0.77663
H	37	0.21464	0.00000	0.78408	0.00128	0.78536
H	38	0.20486	0.00000	0.79378	0.00137	0.79514
H	39	0.21145	0.00000	0.78711	0.00144	0.78855
H	40	0.21144	0.00000	0.78720	0.00135	0.78856
H	41	0.20825	0.00000	0.79055	0.00120	0.79175
H	42	0.22336	0.00000	0.77516	0.00149	0.77664
H	43	0.21464	0.00000	0.78408	0.00128	0.78536
H	44	0.20486	0.00000	0.79377	0.00137	0.79514
H	45	0.21145	0.00000	0.78710	0.00144	0.78855
H	46	0.21144	0.00000	0.78720	0.00135	0.78856
H	47	0.20285	0.00000	0.79557	0.00159	0.79715
C	48	-0.20591	1.99914	4.18860	0.01817	6.20591
H	49	0.20286	0.00000	0.79555	0.00159	0.79714
C	50	-0.20591	1.99914	4.18859	0.01817	6.20591
H	51	0.20751	0.00000	0.79093	0.00157	0.79249
C	52	-0.20328	1.99915	4.18591	0.01822	6.20328
H	53	0.20751	0.00000	0.79093	0.00157	0.79249
H	54	0.20775	0.00000	0.79080	0.00144	0.79225
* Total *		0.00000	55.97543	141.46153	0.56303	198.00000

Supplementary Methods

Crystallographic Methods

X-ray data collection and structural refinement. Intensity data for compounds **3**, **4**, **5**, **6**, and **7** were collected using a Bruker APEX II diffractometer. The crystals of **4**, **5**, **6** and **7** were measured at 103(2) K and the crystals of **3** were measured at 296(2) K. The structure was solved by direct phase determination (SHELXS-97)[1] and refined for all data by full-matrix least squares methods on F^2.[2] All non-hydrogen atoms were subjected to anisotropic refinement. The hydrogen atoms were generated geometrically and allowed to ride in their respective parent atoms; they were assigned appropriate isotropic thermal parameters and included in the structure-factor calculations. CCDC:1049275-1049279 contains the supplementary crystallographic data for this paper. The data can be obtained free of charge from the Cambridge Crystallography Data Center via www.ccdc.cam.ac.uk/data_request/cif.

Computational Methods

Gaussian 09 was used for all density functional theory (DFT) calculations[3] including geometry optimization, frequency calculations, Natural bond orbital (NBO) analysis, and nucleus-independent chemical-shift (NICS) calculations. All calculations were performed at the B3LYP/6-311+G(d,p) level of theory.

Supplementary References

1. G. M. Sheldrick, SHELXL-97, Program for Crystal Structure Refinement; University of Göttingen: Göttingen, Germany, **1997**.
2. Bruker AXS SHELXTL, Madison, WI; SHELX-97G. M. Sheldrick, *Acta Crystallogr. A*, **2008**, *64*, 112–122, *SHELX-2013*, http://shelx.uni-ac.gwdg.de/SHELX/index.php.
3. Gaussian 09, Revision B.01, M. J. Frisch, G. W. Trucks, H. B. Schlegel, G. E. Scuseria, M. A. Robb, J. R. Cheeseman, G. Scalmani, V. Barone, B. Mennucci, G. A. Petersson, H. Nakatsuji, M. Caricato, X. Li, H. P. Hratchian, A. F. Izmaylov, J. Bloino, G. Zheng, J. L. Sonnenberg, M. Hada, M. Ehara, K. Toyota, R. Fukuda, J.Hasegawa, M. Ishida, T. Nakajima, Y. Honda, O. Kitao, H. Nakai, T. Vreven, J. A.Montgomery, Jr., J. E. Peralta, F. Ogliaro, M. Bearpark, J. J. Heyd, E. Brothers, K.N.Kudin, V. N. Staroverov, T. Keith, R. Kobayashi, J. Normand, K. Raghavachari, A. Rendell, J. C. Burant, S. S. Iyengar, J. Tomasi, M. Cossi, N. Rega, J. M. Millam, M. Klene, J. E. Knox, J. B. Cross, V. Bakken, C. Adamo, J. Jaramillo, R. Gomperts,R. E. Stratmann, O. Yazyev, A. J. Austin, R. Cammi, C. Pomelli, J. W. Ochterski, R. L. Martin, K. Morokuma, V. G. Zakrzewski, G. A. Voth, P. Salvador, J. J.Dannenberg, S. Dapprich, A. D. Daniels, O. Farkas, J. B. Foresman, J. V. Ortiz, J.Cioslowski, D. J. Fox, Gaussian, Inc., Wallingford CT, **2010**.
